# Plant extract-mediated green-synthesized CuO nanoparticles for environmental and microbial remediation: a review covering basic understandings to mechanistic study

**DOI:** 10.1039/d5na00035a

**Published:** 2025-03-19

**Authors:** Mashrafi Bin Mobarak, MD. Foysal Sikder, Khandakar Sidratul Muntaha, Shariful Islam, S. M. Fazle Rabbi, Fariha Chowdhury

**Affiliations:** a Institute of Glass and Ceramic Research and Testing (IGCRT), Bangladesh Council of Scientific and Industrial Research (BCSIR) Dhaka 1205 Bangladesh mashrafibinmobarak@gmail.com; b Department of Applied Chemistry and Chemical Engineering, Bangabandhu Sheikh Mujibur Rahman Science and Technology University Gopalganj 8100 Bangladesh; c Biomedical and Toxicological Research Institute (BTRI), Bangladesh Council of Scientific and Industrial Research (BCSIR) Dhaka 1205 Bangladesh chowdhuryfariha@gmail.com

## Abstract

This review provides a comprehensive overview of nanoparticles, with a particular focus on plant extract-mediated green-synthesized copper oxide nanoparticles (CuO NPs). This article is one of the simplest to read as it aims at beginner researchers, who may not have advanced knowledge on topics like nanoparticles, including metal and metal oxide nanoparticles, their classification, and techniques to prepare them. Various synthesis procedures are discussed, emphasizing green synthesis methods that utilize plant extracts as reducing and stabilizing agents. Subsequently, the mechanisms involved in the formation of CuO NPs are highlighted. Their significant applications with a mechanistic overview on environmental remediation, especially in the eradication of textile dyes and pharmaceutical wastes, and their antimicrobial properties are elucidated. By carefully scrutinizing the information available in the literature, this article aims to equip novice researchers with a foundational understanding of nanoparticles, their synthesis, and their practical applications, fostering further exploration in the field of nanotechnology.

## Abstract 2

Environmental and microbial remediations using green-synthesized CuO nanoparticles from plant extracts.

## Graphical abstract

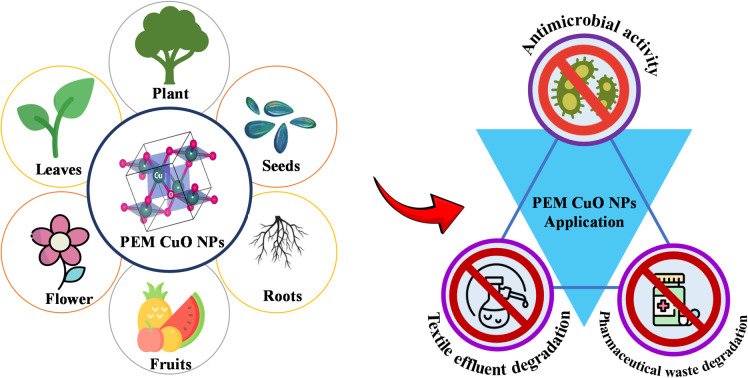

## 1. Introduction

Research on nanomaterials, which have at least one dimension within the nanoscale region (1–100 nm), known as nanomaterials, has become one of the most intriguing disciplines of science. As the surface areas of nanomaterials becomes larger compared to their size, they exhibit superior physical and chemical properties compared with their bulk counterparts.^1^ From electronics to medicine, nanomaterials have been offering enhanced performance, efficiency, and functionality, enabling targeted drug delivery, improved energy storage, and production of light-weight materials with a higher strength.^2^ Among various types of nanomaterials, transition metal oxide (MO) NPs have garnered significant attention owing to their comprehensive applicability in functional smart material fabrication.^3^ The MO NPs beget unique phenomena such as quantum confinement effects, altered surface energies, interface effects, and increased surface-to-volume ratios, which are not observed in non-NPs of MO.^4^

Copper(ii) oxide (CuO) has gained great interest in recent years as a p-type semiconductor with its monoclinic structure, superior conductivity, photovoltaic features, and large stability among nanomaterials.^5^ Realizing and utilizing these exceptional properties broadened the scope of applications of CuO NPs as effective anti-bacterial and anti-fungal agents,^6,7^ materials for environmental remediation *via* photodegradation or adsorptive removal of toxic substances,^8,9^ catalysts for increasing the rate of oxidation reactions,^10^ sensors for the identification of gases and biomolecules,^11,12^ and energy-storing materials, such as batteries or supercapacitors with the desirable electrochemical characteristics.^13^

There are several existing methods widely used for the synthesis of CuO NPs, which include chemical precipitation, sol–gel, hydrothermal, and biological methods.^14^ The traditional chemical and physical methods of CuO NP synthesis often involve toxic reagents and complex procedures. To reduce or eliminate the use of toxic chemicals, finding an alternative source is crucial, and the use of plant extracts for the green synthesis of CuO NPs presents a sustainable alternative.^15^ In this greener approach, the phytochemicals that are present in the plant extract act as a reducing and stabilizing agent for the CuO NPs. Through this process, use of harmful chemicals can be avoided, and thus, an eco-friendly synthesis procedure can be established. Various plant sources such as *Averrhoa carambola*,^6^*Psidium guajava*,^16^*Catha edulis*,^17^ and *Moringa oleifera*^18^ have been exploited for this purpose.

The process through which CuO NPs are synthesized using plant extract is a very intricate method, as it involves several biochemical interactions. The biomolecules from the plant extract facilitate the conversion of the metal precursor into the desired NPs by acting as reducing agents. Additionally, they also stabilize the formed NPs by preventing agglomeration. This process not only yields NPs with controlled size and morphology but also enhances their functional properties.^19^ The plant extract-mediated synthesized CuO NPs exhibit significant potential in environmental applications, particularly in the photodegradation and adsorptive removal of textile dyes and pharmaceutical wastes.^15^ The narrow band gap of CuO NPs, which is typically around 2.1 to 2.71 eV, is beneficial since it enables efficient charge separation and a high redox potential. These features coupled with a large surface area allow the CuO NPs to efficiently photocatalyze the complex organic pollutants and decompose them into less harmful by-products through advanced oxidation processes.^20^ Studies have demonstrated that CuO NPs can effectively degrade pollutants by generating reactive oxygen species (ROS) which react with pollutant molecules, leading to their mineralization.^21^ Furthermore, the plant extract-mediated CuO NPs have shown promising antimicrobial properties against a range of bacterial and fungal strains.^22^ The mechanism of CuO NP's antimicrobial activity mainly involves the disruption of microbial cell wall and the generation of ROS that induce oxidative stress within the microbial cell.^14^ This dual functionality, environmental remediation and antimicrobial activity make plant extract-mediated CuO NPs stand out as a valuable material in both wastewater treatment and healthcare applications.

The present review seeks to give a detailed account of the biogenic synthesis of CuO NPs *via* plant extracts, starting with the fundamentals of the NPs and going further to explore their synthesis, degradation/adsorptive capabilities and antimicrobial properties. Starting with the latest research enhanced with fundamental principles, this article emphasizes the opportunities that green nanotechnology could provide for the NP synthesis as a way of achieving environmental sustainability.

## 2. Basics into nanoparticles

The prefix “nano” originates from the Greek word for “dwarf” and signifies one billionth (10^−9^) of a unit. Although there are various definitions for NPs, to date, a single and universally accepted definition is not yet established.^23^ The International Union of Pure and Applied Chemistry (IUPAC) defines a NP as “*a particle of any shape with dimensions ranging from 1 to 100 nanometers (nm) (1 nm = 1 × 10*^*−9*^*meters)*”.^24,25^ The American Society for Testing Materials (ASTM) offers a slightly broader definition: “*a particle with at least one dimension larger than 1 nanometer and smaller than 100 nanometers, and which may or may not exhibit properties that are dependent on its size*”.^26^ Mainly there are two common definitions for NP size. One definition specifies a diameter range of 1–100 nm.^27^ To help visualize this scale, a human hair is roughly 60 000 nm thick, while an atom has a radius of about 0.1 nm, and a DNA double helix has a radius of 2 nm.^23^ Particles smaller than 1 nm are typically referred to as atomic clusters. Interestingly, some particles larger than 100 nm can still exhibit properties associated with NPs.^28^

Richard P. Feynman, the American physicist, envisioned the idea of nanotechnology and is regarded as the “father of nanotechnology”. In one of his famous talks “*There's Plenty of Room at the Bottom*,” in 1959, Feynman famously posed the question, “Why cannot we write the entire 24 volumes of the Encyclopedia Britannica on the head of a pin?” which initiated the concept of nanotechnology.^23,29^ The existence of NPs is dependent on the chemical and electromagnetic properties of the material and the forms in which they exist include aerosols (solid or liquid particles suspended in air), suspensions (solid particles dispersed in liquids), and emulsions (mixtures of two immiscible liquids).^30,31^ The structure of NPs can range from simple and uniform to complex and layered, depending on whether they are composed of a single material or a combination of materials. Complex NPs may have multiple layers including a surface layer, a shell, and a core.^32^

### 2.1. Impact on properties for nano-size

NPs exhibit distinct physicochemical properties from their bulk counterparts of the same material.^33^ These unique properties stem from two primary reasons: surface effects and quantum confinement.^29^ As the particle size decreases, the number of surface atoms significantly increases out of the total number of particles that contributes towards the geometry. This increase in surface area is extremely significant for determining several properties. Another reason is that, at the nanoscale, the movement of electrons is confined to distinct energy states as a result of confinement into a restricted volume. This effect, known as quantum confinement, modifies the electronic, optical, and magnetic properties of the material.^34^ The effects on other properties include improved reactivity, catalysis, and heat conductivity when the particle size is reduced to the nanoscale. A larger amount of surface electrons facilitate heat transfer, increasing the thermal conductivity. Smaller NPs show a lower melting point, attributed again to surface effects. Since NPs possess more of the surface atoms, they have a proportionately higher surface energy, resulting in their higher chemical reactivity. This would increase their reactivity as effective catalysts, with smaller NPs being more catalytic.^35^

Metallic and semiconductor NPs display unique optical properties for two primary reasons: quantum confinement and localized surface plasmon resonance (LSPR). The latter phenomenon arises from the collective oscillation of electrons on the NP surface when the NP is excited by light. This phenomenon affects the interaction of light with the NP.^36–38^ The energy difference between a material's valence band (filled electron states) and conduction band (empty electron states) is termed the band gap. In NPs, the band gap typically increases compared to bulk materials. This altered band gap affects how light interacts with the NP, leading to different absorption and emission behaviors.^39^ The increased band gap in NPs can also influence electrical properties by affecting the mobility of charge carriers (electrons and holes). In some metallic NPs, a higher band gap can lead to a decrease in electrical conductivity. For instance, Cu NPs may lose conductivity at certain sizes, while conversely, insulating materials such as SiO_2_ can exhibit some degree of conductivity at the nanoscale. The uneven distribution of electrons in some NPs can induce magnetic properties.^35^ Additionally, the large surface-to-volume ratio of NPs can influence the magnetic coupling between neighboring particles, affecting their overall magnetic behavior. The size and shape are also crucial factors in determining the magnetic properties of NPs.^31^

### 2.2. Classification of nanomaterials

The classification of nanomaterials can be done based on their origin, dimensionality (number of dimensions within the nanoscale) and chemical composition of the nanostructures.^35,40,41^

#### 2.2.1. Classification based on origin

Nanomaterials can be divided into two classes based on their origin: natural and synthetic nanomaterials.

##### 2.2.1.1. Natural nanomaterials

Nanomaterials that are formed through natural processes and found in nature fall into this category. The examples are volcanic ash, viruses, protein structures, wings of insects, spider silk, milk and blood which is natural colloid, and ocean spray.^41^

##### 2.2.1.2 Synthetic nanomaterials

Synthetic or artificial nanomaterials are engineered in laboratory or industrial settings to achieve desired morphologies and functionalities. The formation of these NPs is achieved by various processes such as chemical synthesis, physical vapor deposition and self-assembly. The examples of synthetic nanomaterials are quantum dots, carbon-based materials such as graphene and polymeric NPs.^29,41^

#### 2.2.2. Classification based on dimension

Nanomaterials can be classified into four categories based on their dimension: 0D, 1D, 2D and 3D.

##### 2.2.2.1. 0D (zero-dimensional)

When all three dimensions (*x*, *y* and *z*) of a material are confined to the nanoscale, it is termed 0D material and is falls under this category. The examples are fullerene which is a tiny spherical cage made entirely of carbon.

##### 2.2.2.2. 1D (one-dimensional)

Materials with one dimension extending beyond the nanoscale, and other two dimensions within the nanoscale range are termed 1D nanomaterials. Nanowires, nanotubes, nanofibers, and nano rods are examples of 1D nanomaterials.

##### 2.2.2.3. 2D (two-dimensional)

Materials with thin films or plate-like structures which have one dimension in the nanometer range *are* depicted as 2D nanomaterials. Graphene, a single layer of carbon atoms arranged in a honeycomb lattice, is a prime example of a 2D nanomaterial.

##### 2.2.2.4. 3D (three-dimensional)

Materials, whose building blocks or constituents are within the nanometer range, even if the overall dimensions of the material are larger, are considered 3D nanomaterials. These materials are composed of numerous nanosized crystals arranged in various configurations. The examples of this category are bundles of nanowires and nanotubes.

#### 2.2.3. Classification based on chemical composition

Apart from origin and dimension, nanomaterials can be further categorized based on their chemical composition.

##### 2.2.3.1. Carbon-based nanomaterials

The carbon-based nanomaterials are made entirely out of carbon and have so many exceptional properties such as high conductivity (thermal and electrical), extreme strength, and biocompatibility. This category includes fullerenes, graphenes, carbon nanotubes (CNTs), and carbon nanofibers. CNTs are basically rolled-up graphene sheets. They have amazing strength-to-weight ratios.

##### 2.2.3.2. Metal nanomaterials

The subdivision of bulk metals into small assemblies of clusters creates these nanomaterials, which have more novel optical and electrical behaviors because of the quantum confinement effects. Nanomaterials can be synthesized using several metals including gold, silver, and iron.

##### 2.2.3.3. Metal oxide nanomaterials

Metal oxides are formed from their respective metals, and exhibit modified properties, especially improved reactivity and efficiency. They are utilized in supercapacitors, gas sensors, and various solar cell types. Notable examples are CuO, ZnO, NiO, Fe_3_O_4_, and TiO_2_.

##### 2.2.3.4. Organic nanomaterials

Organic nanomaterials consist of organic molecules such as proteins, liposomes, lipids, or polymers. Their biodegradable and non-toxic nature makes them particularly suitable for applications in drug delivery and tissue engineering.

##### 2.2.3.5. Inorganic nanomaterials

This extensive category includes both metal and metal oxide nanomaterials, typically produced by precipitating inorganic salts. Ceramic nanomaterials, recognized for their heat resistance and chemical stability, are a key example and are commonly used in drug delivery.

**Fig. 1:**
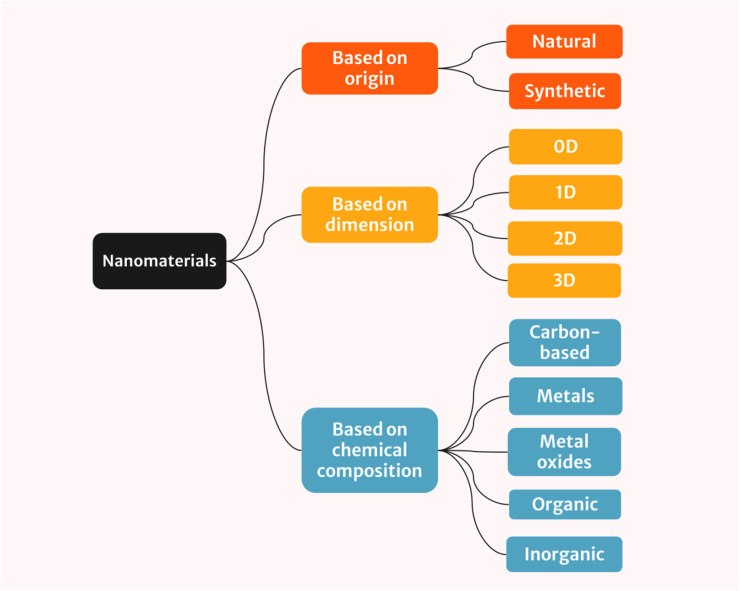
Classification of nanomaterials based on origin, dimension and chemical composition.

The visual representation of nanomaterial classification is shown in Fig. 1.

### 2.3. Synthesis methods of NPs

The synthesis of nanomaterials is a fascinating field which basically has two main approaches: bottom–up and top–down.^29^ Each approach offers unique advantages and is suited for different types of NPs.

#### 2.3.1. Bottom–up approach

This approach, like constructing a miniature building, involves the controlled assembly of atoms or molecules into NPs. It offers precise control over size, composition, and sometimes even shape. There are some techniques that involve such approach for achieving NPs, and some for the widely used techniques are mentioned below.

##### 2.3.1.1. Sol–gel method

This versatile technique involves forming a liquid suspension (sol) containing precursors. These precursors transform into a gel network and eventually into NPs through controlled reactions. It is particularly useful for metal oxides.^42^

##### 2.3.1.2. Precipitation method

This method begins by dissolving a desired metal salt in a solution. By adding another chemical, the dissolved ions come together as solid NPs *via* a precipitation reaction. This method is simple but offers less control over size and shape.^43^

##### 2.3.1.3. Reduction method

This method involves reducing a metal salt to its elemental state using a reducing agent. For example, the Turkevich method (citrate reduction) is a popular way to synthesize spherical gold NPs.^44^

##### 2.3.1.4 Chemical vapor deposition method

Chemical Vapor Deposition, or CVD, is a technique for creating thin films on surfaces. In this process, a heated substrate is exposed to a combination of gaseous chemicals inside a chamber at normal temperature. These chemicals react upon contact with the hot substrate, forming a thin layer of desired material on its surface. The resulting film is then collected for use. The success of CVD depends on the temperature of the substrate. While CVD produces high-quality, uniform, and strong films, it requires specialized equipment and can generate hazardous gaseous byproducts.^45^

##### 2.3.1.5. Spinning method

Spinning disc reactors (SDRs) are one of the important tools for NP creation. Inside the SDR, a disc spins at high speeds, while a liquid precursor and water are pumped in. This fast-paced environment fuses atoms and molecules together forming NPs. The superior feature of SDRs is the tunability; by adjusting factors such as spin speed and liquid flow, the size and properties of the NPS can be controlled.^46^

##### 2.3.1.6. Pyrolysis process

Industrial NP production often relies on pyrolysis, a burning process. Here, a precursor, either liquid or vapor, is fed into a furnace and incinerated at high pressures. The resulting gases are then filtered to collect the NPs. Some variations use lasers or plasma instead of flames for even higher temperatures. Pyrolysis is popular for its simplicity, efficiency, affordability, and ability to continuously produce large quantities of NPs.^47^

##### 2.3.1.7 Molecular condensation method

Molecular condensation, also known as atomic or molecular beam condensation, involves evaporating or sputtering a bulk material in a vacuum chamber to produce a vapor. This vapor then condenses into NPs upon collision with an inert gas like helium or argon. The NP size and morphology can be controlled by adjusting parameters such as gas pressure, flow rate, and temperature. This method is commonly used to produce metallic NPs in a pure, uncontaminated form.^48^

##### 2.3.1.8. Sonochemical synthesis

Sonochemical synthesis uses high-intensity ultrasound to drive chemical reactions and produce NPs. The ultrasound creates cavitation bubbles in a liquid solution that rapidly collapse, generating localized hot spots with extreme temperatures and pressures. This allows for the rapid nucleation and growth of NPs from dissolved precursors. Sonochemical methods can produce a variety of NP compositions including metals, metal oxides, and semiconductors. Key advantages are the ability to control particle size and the mild reaction conditions compared to other techniques.^49^

##### 2.3.1.9. Electrochemical synthesis

Electrochemical synthesis of NPs involves reducing metal ions in solutions at an electrode surface to form NPs. This is done by applying a potential difference between two electrodes immersed in an electrolyte solution containing metal salts. The reduced metal atoms nucleate and grow into NPs on the electrode surface. Parameters such as applied potential, electrolyte composition, and reaction time can be tuned to control the NP size and morphology. Electrochemical methods are simple, cost-effective, and can be scaled up for industrial production of NPs.^50^

##### 2.3.1.10. Biosynthesis

Biosynthesis offers an environment friendly method for creating NPs. This technique utilizes bacteria, plant extracts, and fungi (along with precursors) as a safe alternative to traditional chemicals for reducing and stabilizing the NPs during synthesis. The resulting bio-synthesized NPs possess unique and improved properties, making them well suited for biomedical applications.^51^

#### 2.3.2. Top–down approach

The top–down approach of NP synthesis involves breaking down larger bulk materials into nanoscale particles. This approach is characterized by its destructive nature, where macroscopic structures are reduced to nanometric sizes through various physical processes.^52^ Below is a brief overview of the top–down synthesis methods.

##### 2.3.2.1. Mechanical milling

Mechanical milling offers a cost-effective approach for transforming bulk materials into nanoscale components. This technique excels in creating homogeneous mixtures of different materials, making it particularly useful for producing nanocomposites. Its applications span a wide range including the development of reinforced aluminum alloys, durable coatings, and advanced nanoalloys. Moreover, carbon nanomaterials produced through mechanical milling represent a promising new class of materials with potential for addressing environmental, energy storage, and energy conversion challenges.^53^

##### 2.3.2.2. Lithography

Lithography is a critical technique for constructing nanoscale structures using focused light or particle beams. This method can be categorized into two primary approaches: masked and maskless lithography. Masked lithography involves transferring intricate patterns onto a large surface using a predefined template or mask. The examples of masked lithography are photolithography, nanoimprint lithography, and soft lithography. In contrast, maskless lithography offers greater flexibility by directly writing arbitrary patterns without the need for a mask. This approach encompasses techniques such as scanning probe lithography, focused ion beam lithography, and electron beam lithography. Moreover, by combining focused ion beam implantation with wet chemical etching, it is possible to create complex three-dimensional micro- and nano-structures.^54,55^

##### 2.3.2.3. Laser ablation

Laser ablation is a green synthesis technique that produces NPs by vaporizing a target material with high-energy laser pulses.^56^ This versatile method can create a wide array of nanomaterials including metals, carbon-based structures, and ceramic composites. Unlike traditional methods, it eliminates the need for stabilizing chemicals. Pulsed laser ablation in liquids is particularly promising for producing uniformly sized NP solutions without the use of surfactants. By carefully controlling laser parameters such as energy, wavelength, and the addition of salts, fine-tuning of size and distribution of the resulting NPs is possible.^57,58^

##### 2.3.2.4. Sputtering

Sputtering is a technique for creating NPs by bombarding a solid target with high-energy particles.^59^ This process effectively generates thin films. Energetic gas ions impact the target, dislodging tiny clusters of atoms. The specific configuration of ions and their energy determine the size of these ejected clusters. Sputtering can be achieved through various methods including magnetron, radio-frequency diode, and DC diode sputtering. Typically conducted in a vacuum chamber, sputtering involves introducing a gas and applying a high voltage to the target. This creates ions which collide with the target, ejecting atoms. Magnetron sputtering has been successfully used to produce layered WSe_2_ nanofilms on SiO_2_ and carbon paper substrates. A key advantage of sputtering is its ability to produce NPs with minimal impurities and at a lower cost than that of electron-beam lithography.^60–62^

##### 2.3.2.5. Electrospinning

**Fig. 2:**
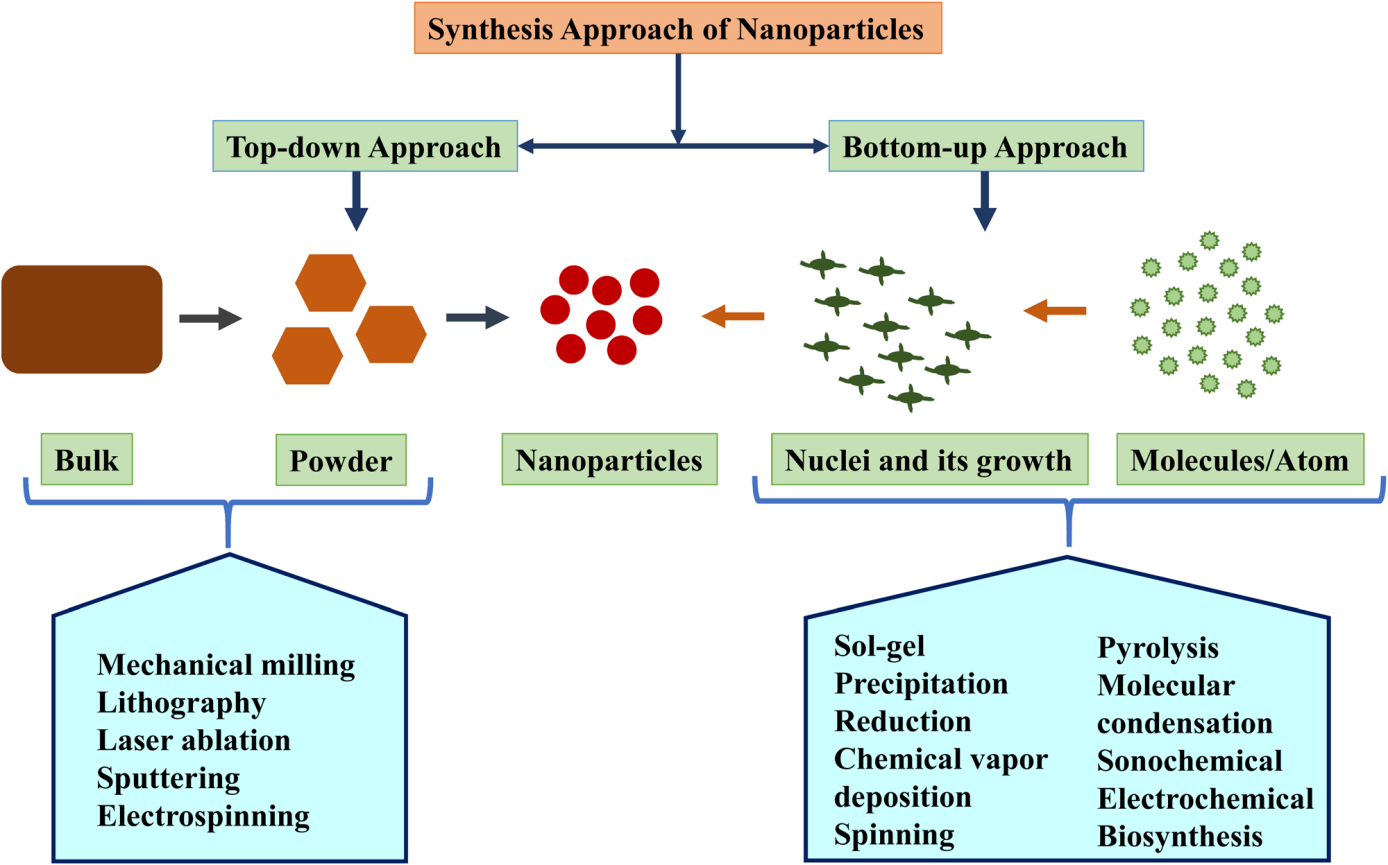
Different approaches for nanoparticle synthesis.

Electrospinning is considered as one of the easiest top–down methods for creating nanostructured materials, particularly nanofibers. It works with a wide range of materials, most commonly polymers. A major innovation in this field is coaxial electrospinning. This technique utilizes a spinneret with two tiny tubes, one inside the other. By pumping different liquids through these tubes, core–shell nanofibers using an electric field can be created. One liquid can be viscous, while the other is less so, allowing for a core-and-shell structure. Coaxial electrospinning shines as a simple and efficient top–down method for producing large quantities of core–shell ultrathin fibers, sometimes reaching centimeters in length. This versatile technique has been used to develop core–shell and hollow materials from various classes including polymers, inorganic materials, organics, and even hybrids.^63–65^Fig. 2 shows the brief overview of the synthesis approaches of NPs.

## 3. Brief introduction of metal oxide NPs

In chemistry, an oxide refers to a compound containing oxygen, bonded to at least one other element. Another way to view this is as a combination of an element and the oxygen ion (O^2−^). In these compounds, oxygen typically has an oxidation state of −2. MOs can be categorized based on the number of oxygen atoms bonded to the metal and the metal's specific oxidation state. Notably, MOs exhibit a wide variety of structures, ranging from simple molecules to complex polymers and crystalline arrangements.^66,67^ Recently, inorganic MO NPs have gained significant interest compared to organic MO NPs. Their high surface area-to-volume ratio, compared to bulk materials, offers several advantages. These advantages include enhanced catalytic activity, exceptional loading capacity, the ease of surface functionalization, applications in drug delivery and biomedicine, and tunable mechanical properties. Among various inorganic nanomaterials (NMs), MO NPs are particularly attractive and widely used due to several factors. First, well-established and simple synthesis methods exist for MO NPs. Second, their physicochemical properties can be easily controlled. Finally, MO NPs possess fascinating and tunable characteristics, making them versatile for various applications.^68^ MO NPs have a wide range of applications across various scientific and medical fields. Some of the key applications include energy storage and conversion (batteries,^69^ supercapacitors,^70^ solar cells,^71^*etc.*), biomedical applications (tissue engineering, cancer treatment, gene therapy, wound healing,^72^*etc.*), environmental and chemical applications (gas sensors,^73^ catalysis,^74^ antibacterial and antifungal applications,^75^*etc.*), electronics and optics (antennas, rectifiers, optoelectronics^76^*etc.*), and resistive switching and magnetic applications (resistive switching devices, magnetic storage devices,^77^*etc.*). These applications highlight the versatility and potential of MO NPs in various fields, from energy and biomedical applications to environmental and electronics fields.

While both metal and metal oxide NPs offer exciting possibilities in nanotechnology, MO NPs possess several advantages that make them a more versatile and tunable platform for various applications. Unlike pure metals, which can be prone to oxidation or degradation in certain environments, MO NPs offers superior chemical stability. This makes them more durable and reliable for long-term applications.^78^ Moreover, the addition of oxygen to a metal introduces a new dimension for manipulating properties. By varying the metal type, oxidation state, and particle size, it is possible to achieve a wider range of electrical, optical, and catalytic properties in MO NPs compared to their pure metal counterparts.^79^ On top of that, MO NPs possess surfaces rich in hydroxyl groups (OH^−^) that readily react with other molecules. This allows for easier surface functionalization, enabling them to be tailored for specific purposes such as drug delivery or binding to target biomolecules.^80^ When it comes to biocompatibility, some MO NPs exhibit better biocompatibility than pure metals. This makes them more suitable for applications in medicine and diagnostics, where interaction with biological systems is crucial.^78^ However, the environmental impact of both types of NPs needs careful evaluation. Some MO NPs is less toxic or easier to manage in the environment than their pure metal counterparts.

The research on MO NPs is constantly evolving, and new applications are being discovered all the time. The MO NPs that are being actively researched include zinc oxide NPs (ZnO NPs), iron oxide NPs (Fe_2_O_3_ and Fe_3_O_4_ NPs), titanium dioxide NPs (TiO_2_ NPs), silicon dioxide NPs (SiO_2_ NPs), copper oxide NPs (CuO NPs), and aluminum oxide NPs (Al_2_O_3_ NPs). These MO NPs have been extensively studied due to their unique properties and potential applications in various fields including biomedicine, environmental remediation, and energy storage.

Zinc oxide (ZnO) NPs are characterized by their wide bandgap, excellent electrical conductivity, and strong antibacterial properties. These attributes make them highly versatile, finding applications in various fields. In electronics, ZnO NPs are employed in the fabrication of transparent conductive films and sensors. Their antibacterial nature has led to their incorporation into wound dressings, textiles, and personal care products. Additionally, ZnO NPs are used in sunscreens due to their ability to block UV radiation.^81,82^

Titanium dioxide (TiO_2_) NPs are another class of MO NPs with remarkable properties and wide-ranging applications. TiO_2_ exists in three primary crystalline structures: anatase, rutile, and brookite. Among these, anatase and rutile are the most commonly used forms. TiO_2_ NPs possess excellent photocatalytic activity, which means they can utilize sunlight to break down pollutants and organic compounds. This property has made them indispensable in environmental remediation and water purification. Additionally, TiO_2_ NPs exhibit high refractive index and opacity, making them ideal pigments for paints, coatings, and plastics. Their non-toxicity and UV-blocking capabilities have also led to their extensive use in sunscreens and cosmetics.^83,84^

Iron oxide NPs, primarily in the forms of magnetite (Fe_3_O_4_) and hematite (Fe_2_O_3_), have garnered significant attention due to their magnetic properties. These NPs exhibit superparamagnetism, meaning they can be easily magnetized and demagnetized. This characteristic makes them invaluable in various biomedical applications including drug delivery, magnetic resonance imaging (MRI) contrast agents, and hyperthermia treatment for cancer. Beyond biomedicine, iron oxide NPs find applications in catalysis, environmental remediation, and data storage. Their magnetic properties also enable their use in sensors and actuators.^85,86^

Aluminum oxide (Al_2_O_3_), commonly known as alumina, is a versatile material that exists in various crystalline forms, with the most common being α-alumina. When reduced to the nanoscale, it exhibits unique properties that make it highly desirable for numerous applications. Al_2_O_3_ NPs possess exceptional hardness, a high melting point, excellent chemical stability, and remarkable electrical insulation properties. These characteristics make them ideal for applications in ceramics, abrasives, catalysts, and as reinforcing agents in composite materials. Additionally, alumina NPs have shown promise in biomedical fields due to their biocompatibility and potential for drug delivery.^87–89^

Silicon dioxide (SiO_2_), or silica, is another widely used material, with its nanoscale counterpart offering distinct advantages. SiO_2_ NPs are characterized by their large surface area, high porosity, and excellent thermal stability. These properties make them invaluable in various industries. In the electronics sector, silica NPs are essential components of semiconductors, optical fibers, and sensors. Their biocompatibility and non-toxicity have led to their application in drug delivery systems, biosensors, and tissue engineering. Moreover, silica NPs find use in catalysis, water treatment, and as additives in cosmetics and personal care products.^90–92^

Copper oxide NPs, specifically cupric oxide (CuO), exhibit interesting properties that have attracted significant research interest. They possess excellent electrical conductivity, high catalytic activity, and antimicrobial properties. These characteristics make CuO NPs promising candidates for various applications. In the energy sector, they are employed in lithium-ion batteries, solar cells, and fuel cells. Their catalytic properties find use in environmental remediation, such as the degradation of pollutants. Additionally, CuO NPs have shown potential as antibacterial agents for wound healing and water purification.^9,14,93^ In the following part of this review, details regarding the CuO NPs, their plant extract-mediated green synthesis, application and mechanism are discussed elaborately.

## 4. CuO NPs: the material of interest

CuO NPs have emerged as a captivating subject in materials science due to their exceptional properties as p-type semiconductors with a narrow band gap. Unlike their bulk counterparts, CuO NPs exhibit remarkable physical and chemical characteristics attributed to their large surface area and size-dependent effects.^94,95^ This has sparked intense research interest in their potential applications across diverse fields. CuO NPs are particularly promising as electrode materials for next-generation lithium-ion batteries (LIBs) owing to their high theoretical capacity, safety, and environmental friendliness.^96,97^ Their strong light absorption, low heat emission, and favorable electrical properties make them suitable for solar cell fabrication. Additionally, these nanostructures excel in various applications, including gas and biosensing, nanofluids, photodetection, energy storage, environmental remediation, and catalysis.^5,98^ The versatility of CuO NPs extends to the creation of organic–inorganic nanocomposites with enhanced properties such as thermal and electrical conductivity, mechanical strength, and durability. They effectively catalyze the oxidation of harmful gases and organic compounds, and their superhydrophobic nature shows promise in self-cleaning coatings, water treatment, and oil–water separation. To harness the full potential of CuO NPs, researchers have developed methods to synthesize nanostructures in various shapes and sizes, from zero-dimensional NPs to complex three-dimensional architectures.^99–101^ While cuprous oxide (Cu_2_O) shares some similarities, its distinct properties and lower stability compared to CuO make the latter more suitable for practical applications. Compared to other MO NPs, CuO NPs offer unique advantages in magnetism, superhydrophobicity, and catalysis. Although its application in LIBs is less explored than some counterparts, its favorable attributes make it a promising candidate.^102,103^

### 4.1 Crystal structure of CuO NPs

**Fig. 3:**
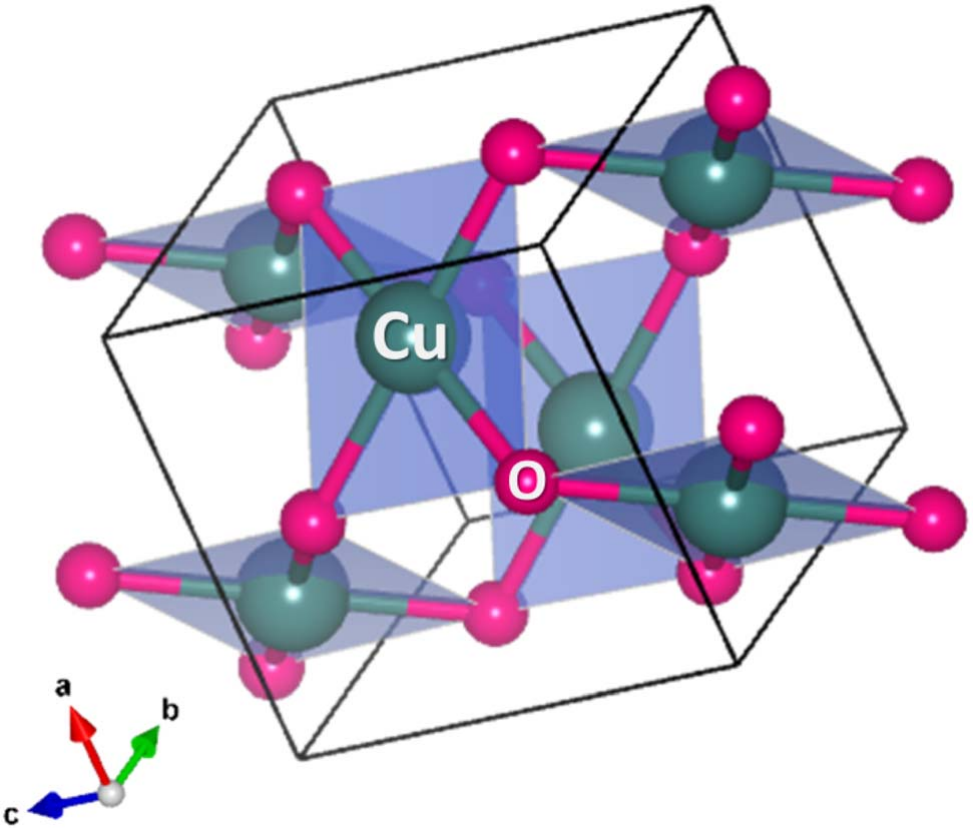
Typical crystal structure of monoclinic CuO (CIF file was taken from Materials Project).

CuO has a distinct crystal structure characterized by its monoclinic symmetry. The crystal system belongs to the space group *C*2/*c*, and its unit cell parameters have been refined through various structural analyses.^104^ In the CuO crystal structure, each Cu atom is coordinated by four O atoms. This arrangement forms a nearly square planar configuration, where the Cu atom is at the center and the O atoms are located at the corners of a distorted tetrahedron. The coordination geometry leads to the formation of ribbons of parallelograms in the [110] direction, while two types of –Cu–O–Cu– chains can be observed in the [101] direction.^105^ CuO exhibits a characteristic structural distortion due to the strong Jahn-Teller effect commonly observed in divalent copper compounds. This distortion results in a square planar arrangement of oxygen atoms around the copper ion. The Cu–O bond distances within this plane are slightly longer than those found in cuprous oxide. The remaining Cu–O distances perpendicular to this plane are significantly longer, definitively excluding an octahedral coordination geometry. Oxygen atoms in CuO are coordinated to four copper atoms in a distorted tetrahedral configuration. While the copper ion in CuO is undeniably in the +2 oxidation state, the bonding within the compound is generally accepted to be a combination of ionic and covalent characters.^5^ The crystallographic parameters of a typical CuO compound are presented in Table 1 and a crystal structure was drawn (Fig. 3) based on the presented crystallographic information using the VESTA version 3 software.^106,107^

**Table 1:** General information and crystallographic parameters of a typical CuO NP sample

Sl. no.	Crystallographic parameter	ICDD card no #00-041-0254
1	Mineral name	Tenorite
2	Crystal system	Monoclinic
3	Space group	*C*2/*c*
4	Space group number	15
5	Unit cell parameters	*a* = 4.6850 Å, *b* = 3.4230, *c* = 5.1320 Å, *α* = *γ* = 90°, *β* = 99.52°
6	Calculated density	6.52 g cm^−3^
7	Measured density	6.45 g cm^−3^
8	Volume of unit cell	81.17 Å^3^
9	Number of symmetry-independent molecules (*Z*)	4.00
10	Reference intensity ratio (RIR)	2.80

### 4.2 Synthesis of CuO NPs

The synthesis of CuO NPs can be broadly classified into three main categories: physical, chemical, and biological synthesis.^108^ The fundamentals of these synthesis categories have been described in the “2.3. Synthesis methods of NPs” section.

#### 4.2.1 Physical synthesis methods of CuO NPs

The physical synthesis methods employ the top–down approach, where bulk materials are decomposed into smaller ones, consequently transforming them into NPs. These methods produce pure nanoproducts but require expensive instruments and high energy. Some physical techniques used for CuO NPs synthesis include ball milling, laser ablation, sputtering, and thermal decomposition. Ball milling is a mechanical process that involves the grinding of materials in a rotating cylindrical chamber filled with balls. The balls transfer their kinetic energy to the material, resulting in particle size reduction and the formation of NPs. For CuO NPs, the process utilizes mechanical alloying, where repeated impacts and friction from the balls cause the CuO precursor to fracture and deform, resulting in NP formation. Factors such as ball-to-powder ratio, milling speed, and time significantly influence the size and morphology of the NPs. This is a cost-effective and simple setup that is capable of producing large quantities of NPs with a high degree of crystallinity and a narrow particle size distribution.^109^ Ayoman *et al.* synthesized CuO NPs through high-energy ball milling for 40 h at room temperature, which resulted in 31 nm-sized particles.^110^ In laser ablation, a high-power laser beam is focused on a copper target, vaporizing the material. The resulting vapor condenses to form CuO NPs.^111^ This method offers high purity and uniformity, and precise control over particle size and morphology by adjusting laser parameters, but it is expensive with limited scalability and requires specialized equipment.^112,113^ The CuO NPs synthesized by pulsed Nd:YAG laser ablation resulted in NPs of 3–40 nm size range measured by TEM analysis.^111^ Ligand-free CuO NPs with a particle size of 35 ± 12 nm were prepared by Maria Censabella *et al.*^114^ Sputtering is a physical vapor deposition technique that involves bombardment of a copper target with ions, causing copper atoms to be ejected and deposited onto a substrate. The deposited copper atoms can then be oxidized to form CuO NPs. This method offers precise control over the NP size and shape, but it can be expensive and requires specialized equipment.^115^ Recently, the reactive magnetron sputtering method has gained much attention due to its efficacy in producing CuO NPs with higher purity and narrower size distribution. In the work of Verma *et al.*,^116^ CuO NPs were synthesized by following the reactive magnetron sputtering technique at different sputtering pressures. Based on the TEM image analysis, CuO NPs of 6 nm size were achieved at 10 mTorr. Thermal decomposition involves heating copper precursor compounds such as copper acetate, oxalate, and nitrate to decompose and form CuO NPs. The decomposition temperature and reaction conditions can influence the NP size, shape, and crystallinity. This technique is relatively simple and inexpensive, but less precise in controlling the NP properties than sputtering.^117,118^

#### 4.2.2 Chemical synthesis methods of CuO NPs

There are several chemical methods that are used to synthesize CuO NPs. These methods typically involve a bottom–up approach where basic units assemble into larger structures to form the NPs. Some of the most common chemical synthesis techniques for CuO NPs include sol–gel, co-precipitation, hydrothermal, solvothermal, microwave-assisted synthesis, and relevant other methods.^22^

The sol–gel method is a popular choice for synthesizing NPs due to its simplicity and efficiency. It offers precise control over particle size, making it ideal for applications requiring specific dimensions. Research has shown that the sol–gel method can produce CuO NPs with sizes ranging from 10 to 100 nm.^119^ Wang *et al.* synthesized CuO NPs with a size of ∼100 nm following the sol–gel technique and investigated the gas sensing efficacy of the synthesized NPs.^120^ The calcination time and temperature play crucial roles in determining the physical properties of CuO NPs synthesized by the sol–gel method. By carefully adjusting these parameters, NP characteristics can be fine-tuned. For instance, Jayaprakash *et al.* successfully synthesized both uncapped and capped CuO NPs using ethylene diaminetetraacetic acid (EDTA) as a capping agent. The capping agent helped control the size and shape of the NPs, demonstrating the versatility of the sol–gel method.^121^

Co-precipitation is a widely used technique for the synthesis of CuO NPs. It involves the simultaneous precipitation of copper and oxygen ions from a solution, leading to the formation of CuO NPs. Different sized and shaped CuO NPs have been prepared using various copper precursors such as copper(ii) nitrate, copper(ii) sulfate, copper(ii) chloride, and copper(ii) acetate.^43,122^ Capping agents, often used as both reducing and stabilizing agents, are typically introduced alongside the precursor materials at the beginning of the chemical synthesis process for various NPs.^123^

The hydrothermal/solvothermal method involves heating a reaction mixture containing copper precursors and a solvent (typically water or an organic solvent) in a sealed autoclave under high-temperature and -pressure conditions. The elevated conditions facilitate the dissolution of the precursors, promote nucleation, and control the growth of the CuO NPs. Hydrothermal/solvothermal synthesis offers several advantages for the preparation of CuO NPs, such as controlled particle size and morphology, high purity, crystallinity, and uniformity. Zhang *et al.* synthesized CuO nanoplatelets following a simple hydrothermal technique where the thickness of the NPs was 65–80 nm.^124^ In the work of Zhao *et al.*, nanosheets of CuO were synthesized in large amounts using the hydrothermal technique, and the thickness of the sheets ranged from 40 to 50 nm.^125^ CuO microspheres consisting of nanosheets were prepared by Wang *et al.* following the solvothermal method, which also included annealing after the synthesis.^126^ Gopalakrishnan *et al.* reported the synthesis of 1D CuO NPs through a surfactant- and template-free solvothermal technique that resulted in nanowires of 15 nm diameter and 90 nm length.^127^

Microwave-assisted synthesis utilizes electromagnetic waves to heat the reaction mixture directly, leading to faster reaction times and improved control over particle size and morphology compared to traditional heating methods. As the polar molecules present in the reaction mixture absorb the microwave energy, they experience rapid oscillations, leading to localized heating and enhanced molecular motion. This increased kinetic energy facilitates the nucleation and growth of CuO NPs, resulting in smaller, more uniform particles with desirable properties. Furthermore, microwave-assisted synthesis often produces NPs with unique properties compared to those synthesized using conventional methods. The rapid heating and localized energy deposition can influence the nucleation and growth kinetics, resulting in particles with different sizes, shapes, and surface morphologies. These properties can have significant implications for the performance of CuO NPs in various applications such as catalysis, sensing, and energy storage.^128^ Different morphologies of CuO were achieved by changing the alkali source by Jung *et al.* following the microwave-assisted synthesis procedure.^129^ Feather- and flower-like CuO nanocrystals were prepared by Zhang *et al.* adapting the microwave-assisted synthesis technique.^130^

#### 4.2.3 Biological synthesis methods of CuO NPs

While traditional chemical and physical methods are commonly used for CuO NP synthesis, biological methods have emerged as a more eco-friendly and sustainable approach and therefore are gaining much attention. These green synthesis techniques utilize natural resources such as plants and microbes to produce CuO NPs.

The microbial synthesis of CuO NPs employs certain microorganisms such as bacteria, fungi, and algae. These biological entities produce CuO NPs either extracellularly or intracellularly. While the exact mechanisms of NP formation using biological agents remain unclear, it is believed that specific biomolecules are involved in this process. Additionally, intracellular and extracellular NP syntheses differ, with the cell wall of microorganisms likely playing a crucial role in the former and extracellular enzymes in the latter. Due to its faster production rate and simpler synthesis process, extracellular NP synthesis has become more prevalent than intracellular methods.^131^ Singh *et al.* synthesized CuO NPs by exploiting^132^*Escherichia coli* (*E. coli*). A report from Ghorbani *et al.* depicts the formation of CuO NPs of size averaging 49 nm, achieved through the extracellular synthesis by *Salmonella typhimurium*.^133^ Chilean white-rot fungus (*Stereum hirsutum*) was used by Cuevas *et al.* for extracellular biosynthesis of spherical CuO NPs (5 to 20 nm).^134^ Brown seaweed (*Sargassum polycystum*) was utilized for the synthesis of CuO NPs, and its antimicrobial and anticancer activities were examined.^135^

The use of plant extracts for the synthesis of CuO NPs is the most widely accepted and implemented “green synthesis” technique. Using plant extracts to make NPs has advantages over other biological methods such as microbial synthesis. The primary raw materials for PEM-CuO NP synthesis, such as leaves, fruits, flowers and fruit peels, are readily available and abundant in nature. Moreover, these materials often require minimal pre-treatment and relatively straightforward extraction process, which lowers the cost of the synthesis.^136,137^ Furthermore, plant-based synthesis generally avoids harsh chemicals and can utilize agricultural waste, minimizing environmental impact.^138^ In contrast, the production cost of the microbial synthesis method of nanoparticles is higher due to the maintenance of microbial cultures, including media preparation and sterilization, compared to plant extract preparation.^139^ Additionally, genetically modified microorganisms can raise ethical concerns and may be subject to stricter regulations. Moreover, separating and purifying nanoparticles from microbial biomass can be a complex and costly process.

However, the exact composition of plant extracts can vary depending on many factors such as season, geographical location, and plant species, potentially affecting the reproducibility and consistency of nanoparticle synthesis. This variability can make it difficult to achieve precise control over size, shape and uniformity compared to some microbial methods.^140^ However, microbial cultures can be grown in controlled environments, leading to high yields and consistent nanoparticle production, and can be readily scaled up for industrial production using bioreactors. Microorganisms, especially genetically modified ones, can offer greater control over the size, shape, and stability of synthesized nanoparticles, as their metabolic pathways can be manipulated to produce nanoparticles with specific characteristics.

**Fig. 4:**
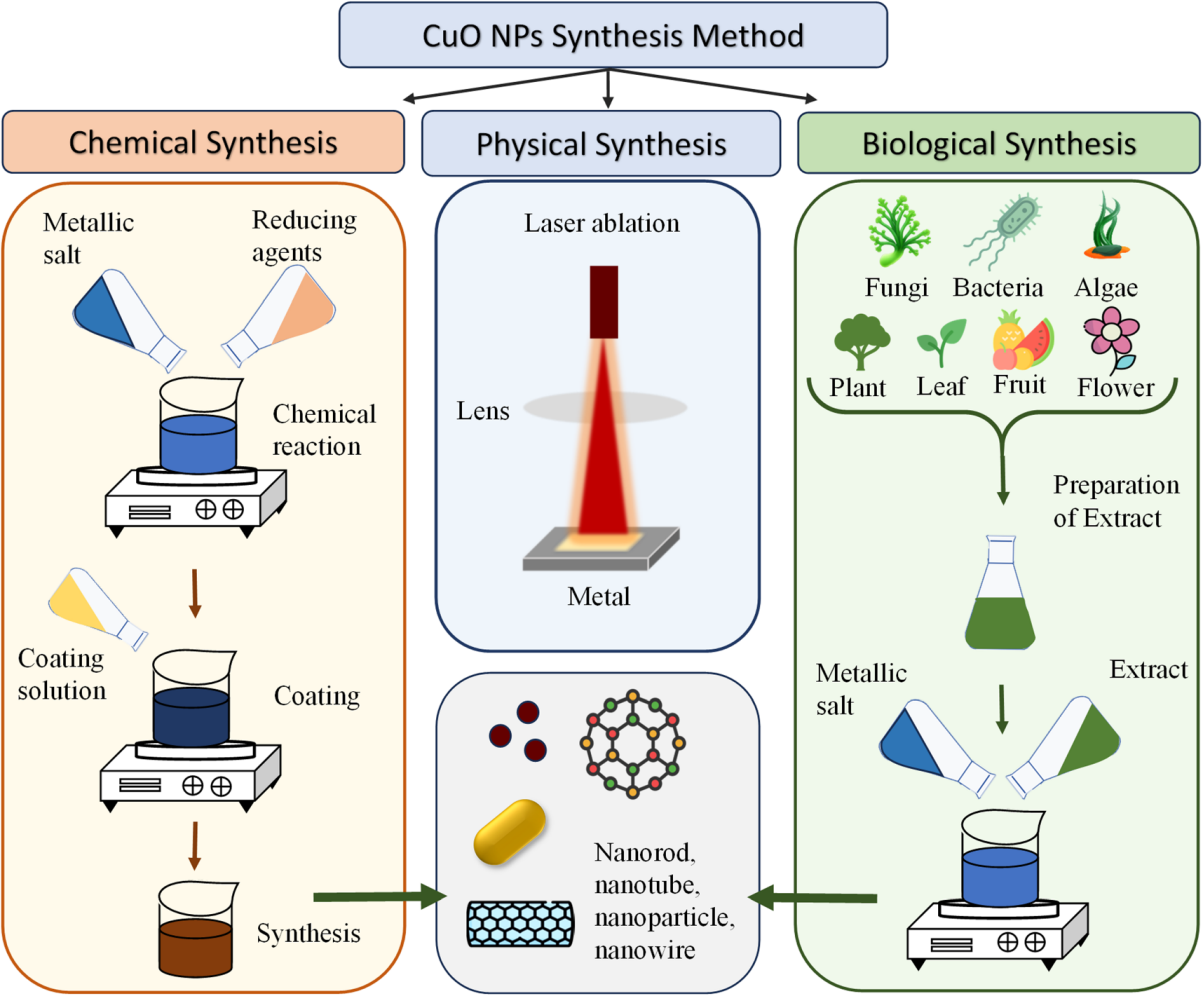
Schematic of the synthesis methodologies of CuO NPs.

Both plant extract-mediated and microbial synthesis offer unique advantages and limitations. On the one hand, plant-based methods are more cost-effective, environmentally friendly and require fewer chemicals. On the other hand, microbial-mediated synthesis offers greater control over nanoparticle properties, but they come with higher costs and potential ethical considerations. The choice between these methods depends on the specific requirements of the application, including the desired nanoparticle properties, production scale, cost constraints, and environmental considerations. However, considering the popularity and widespread use of plant extract-mediated synthesis, this review aims to discuss the implementation of the synthesis of CuO NPs using plant extracts, and hence, detailed discussions are presented in the following section. Fig. 4 graphically summarizes the synthesis methods of CuO NPs.

## 5. Plant extract-mediated synthesis of CuO NPs

While both PEM and microorganism-assisted synthesis procedures are considered green methods for producing CuO NPs, PEM synthesis is often more prominently termed “green” due to several factors. Plant extracts offer a more advantageous approach to synthesizing CuO NPs than microorganisms. While the latter can produce NPs, challenges such as toxicity, isolation, and incubation limit their practicality. Plant extracts, however, are non-toxic, readily available, and can complete the NP synthesis process within a few hours at room temperature.^108^ The bioactive compounds including flavonoids, phenols, and terpenoids in plant extracts act as reducing and stabilizing agents, converting metal salts into NPs.^141^ These compounds generate electrons that reduce the metal ions, leading to NP formation.^142^ Plant-based NP synthesis is a simple, safe, and energy-efficient method that produces stable NPs.^143^ Additionally, this method can be scaled up more easily for industrial production, making it suitable for commercial applications.^144^ Furthermore, plants are often considered more natural and environmentally friendly than microorganisms, which makes PEM synthesis more likely to be termed the “greener” approach. Table 2 summarizes the recent reports on plant sources and precursors used for PEM-CuO NP synthesis, along with the resulting size and morphology.

**Table 2:** Summary of plant sources and precursors utilized for PEM-CuO NP synthesis, along with the resulting size and morphology

Sl. no.	Plant source	Plant part	Precursor used	Particle size	Morphology	Ref.
1	*Averrhoa carambola*	Leaf	Copper sulfate pentahydrate	98 ± 26 nm	Irregular, mostly spherical	6
2	*Aloe vera*	Leaf	Copper nitrate	20–30 nm	Spherical	231
3	*Calotropis gigantean*	Leaf	Copper nitrate	20 nm	Spherical	232
4	*Rubus glaucus*	Leaf and fruit	Copper nitrate trihydrate	43.3 and 52.5 nm	Spherical	233
5	*Ixiro coccinea*	Leaf	Copper sulfate pentahydrate	80–110 nm	Spherical	234
6	*Malva sylvestris*	Flower	Copper nitrate trihydrate	26 nm	Spherical	235
7	*Azadirachta indica*	Leaf	Copper acetate tetrahydrate	12 nm	Spherical	236
8	*Eupatorium odoratum*	Leaf	Copper sulfate pentahydrate	—	Spherical	237
9	*Acanthospermum hispidum*	Leaf	Copper sulfate pentahydrate	—	Spherical	237
10	*Albizia lebbeck*	Leaf	Copper sulfate pentahydrate	<100 nm	Spherical	238
11	*Kalopanax pictus*	Leaf	Copper sulfate pentahydrate	26–67 nm	Spherical	239
12	*Rosa sahandina*	Fruit	Copper sulfate pentahydrate	<50 nm	Spherical	240
13	*Hylotelephium telephium*	Flower	Copper nitrate hexahydrate	83 nm	Spherical	241
14	*Pterolobium hexapetalum*	Leaf	Copper sulfate pentahydrate	10–50 nm	Spherical	242
15	*Tabernaemontana divaricate*	Leaf	Copper sulfate solution	48 nm	Spherical	243
16	*Coriandrum sativum*	Seed	Copper chloride	18.2 nm	Irregular	244
17	*Acalypha indica*	Leaf	Copper sulfate pentahydrate	29 nm	Spherical	245
18	*Albizia lebbeck*	Leaf	Copper sulfate pentahydrate	100 nm	Spherical	246
19	*Pterocarpus marsupium*	Wood	Copper sulfate monohydrate	20–50 nm	Spherical	247
20	*Eichhornia crassipes*	Leaf	Copper sulfate	15–30 nm	Spherical	248
21	*Aloe barbadensis*	Leaf	Copper sulfate	15–30 nm	Versatile and spherical	249
22	*Terminalia catappa*	Leaf	Copper sulfate pentahydrate	103–29 nm	Spherical	250
23	Oak	Fruit	Copper nitrate trihydrate	34 nm	Quasi-cubic	251
24	*Beta vulgaris*	Root	Copper sulfate pentahydrate	11.4 to 63.9 nm	Predominantly spherical and irregular	252
25	*Citrofortunella microcarpa*	Leaf	Copper nitrate trihydrate	54–68 nm	Spherical-like	253

### 5.1. Mechanism of CuO formation in PEM synthesis route

Generally, the PEM synthesis of metal oxide NPs can be explained through two primary mechanisms: chelation-based complex formation and bioreduction. Both of the mechanisms were widely discussed in the literature.^145^

According to the chelation mechanism, the bioactive compounds (extracts such as flavonoids, phenols, and other phytochemicals) present in the plant extract coordinate with metal precursor ions and form stable intermediate complexes.^146^ Upon thermal decomposition during calcination, these complexes lead to the formation of metal oxide NPs. Several studies support this mechanism, including the work by Matinise *et al.*, which demonstrated that antioxidants in *Moringa oleifera* leaf extract chelate zinc(ii) ions, forming zinc–organic complexes that subsequently transform into ZnO NPs upon heat treatment. This conclusion was further reinforced by FTIR spectral analysis, which confirmed the presence of bioactive functional groups in the synthesized NPs.^147–149^

However, the bioreduction mechanism suggests that the phytochemicals act as reducing agents that convert the metal ions into their zero-valent states. These reduced metal atoms then react with the dissolved oxygen present in the reaction medium and lead to the formation of metal oxides. Singh *et al.* proposed this route for the synthesis of ZnO quantum dots using *Eclipta alba* leaf extract, wherein zinc acetate was reduced by phytochemicals before reacting with oxygen to form ZnO. Furthermore, this mechanism suggests that plant-derived compounds also aid in NP stabilization by preventing agglomeration.^150^

For the PEM synthesis of CuO NPs, a similar mechanism can be proposed. The phytochemicals present in the extract may either chelate copper ions to form copper–organic complexes that decompose into CuO during calcination or reduce Cu^2+^ to its metallic state, followed by oxidation to CuO. The presence of functional groups from plant metabolites in the synthesized CuO NPs, as identified by spectroscopic analyses, further supports these proposed pathways. Nagore *et al.* have reported the synthesis of CuO NPs using *Polyalthia longifolia*, which involved the formation of a complex between copper ions and phytochemicals to form CuO NPs *via* a nucleation process and this process continued until the NPs obtained a stable size and shape.^151^ Nagajyothi *et al.* synthesized CuO NPs from black beans (*Phaseolus vulgaris*) and proposed that the water in the system produced OH^−^, which reacted with the metal precursor (copper sulfate heptahydrate) to form Cu(OH)_2_. Then, the phytochemicals present in the black bean aqueous extract encapsulated the CuO NPs by reduction.^152^ Aroob *et al.* utilized *Seriphidium oliverianum* leaf extract to synthesize CuO NPs. From the study, they concluded that various functional groups in flavonoids were responsible for the formation of the NPs. The enol flavonoids were converted to keto flavonoids and accordingly reduced the Cu ions by H atoms released in the process.^21^ Alhalili reported the green synthesis of CuO NPs by the leaf extract of *Eucalyptus globulus.* He abridged the synthesis mechanism by concluding that the metal complex formed with the polyphenols in the extract solution, which eventually was reduced to CuO NPs.^19^ From the work of Nagore *et al.*,^151^ Sutradhar *et al.*,^153^ and Veisi *et al.*,^154^ the fact is established that when the reduction of Cu ions produces Cu metal atoms, they are converted to CuO NP by air O_2_ or dissolved oxygen present in the solution at a moderate temperature of approximately 80–85 °C. All of these studies showed that polyol or polyphenols present in the phytochemicals were responsible for the reduction and encapsulation of Cu ions, eventually forming CuO NPs.

**Fig. 5:**
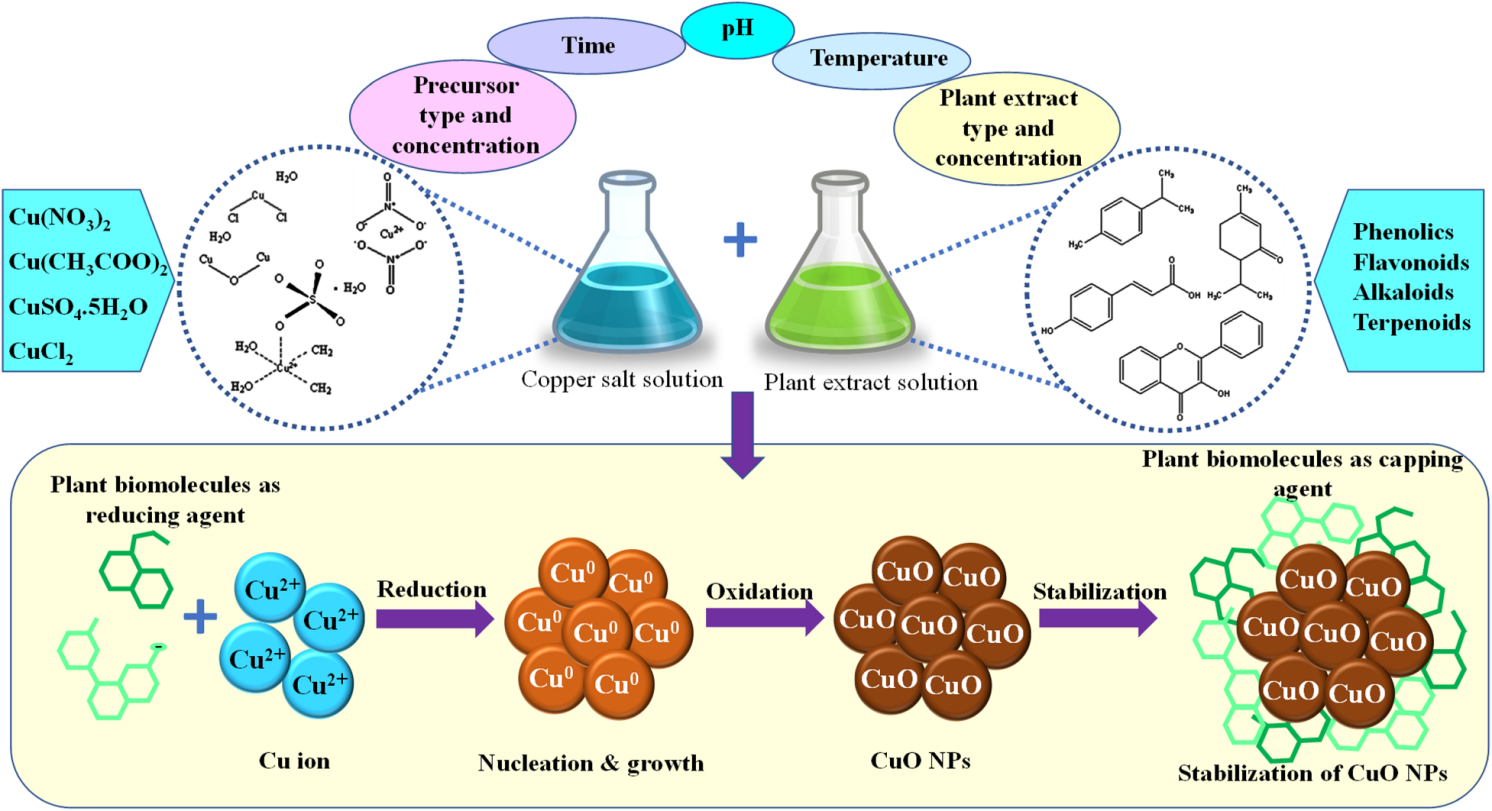
Schematic of the formation mechanism of PEM-CuO NPs.

Based on the concept of chelation mechanism, a generalized reaction scheme of PEM-CuO NPs synthesis can be proposed. Fig. 5 schematically illustrates the formation mechanism of CuO NPs.

Step-1: Chelation of Cu^2+^ by plant extract compoundsCu^2+^ + phytochemicals → Cu-phytochemical complex

For example, if flavonoids (FL) are involved:Cu^2+^ + FL → Cu-FL complex

Step-2: thermal decomposition of Cu-Phytochemical Complex.
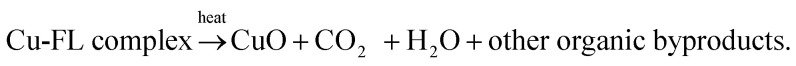


Meanwhile, the basic understanding of bioreduction mechanism aids in creating the reaction scheme as follows.

Step-1: Reduction of Cu^2+^ to Cu^0^ by plant extract compoundsCu^2+^ + phytochemicals → Cu^0^ + oxidized phytochemicals

For example, using ascorbic acid (C_6_H_8_O_6_) as a reducing agent:Cu^2+^ + C_6_H_8_O_6_ → Cu^0^ + C_6_H_6_O_6_ + 2H^+^

Step-2: Oxidation of Cu^0^ to CuO in the presence of oxygen and water.

Reaction with dissolved oxygen in the solution will result in CuO:
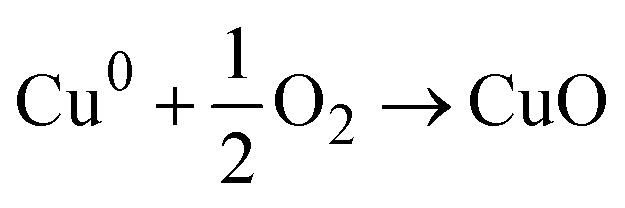


Reaction in the presence of water will result in:
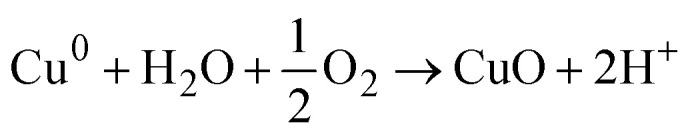


### 5.2. Characterization techniques of CuO NPs

The characterization of CuO NPs is crucial to understand their structural, morphological, and optical properties.^155^ The success of the plant-mediated CuO NP synthesis can be confirmed through the X-ray diffraction (XRD) analysis. XRD provides information about the crystal structure, lattice parameters, and phase purity of the synthesized CuO NPs. It can be used to confirm the formation of the desired CuO phase and identify any impurities, since metallic Cu or Cu_2_O can co-exist with the CuO NPs.^7^ Further clarification or confirmation of CuO formation can be achieved through the X-ray photoelectron spectroscopic (XPS) technique. XPS determines the elemental composition and oxidation states of the elements present in the CuO NPs. It can be used to confirm the presence of copper and oxygen in the desired ratios and to investigate the surface chemistry of the NPs.^6^ In addition to that, the presence of impurities or the elements of capping or stabilizing agents can be detected at the surface level of CuO NPs.

When the formation of CuO phase is confirmed, the next vital characterization would be to carry out transmission electron microscopy (TEM) and/or scanning electron microscopy (SEM). TEM provides high-resolution images of the morphology, size distribution, and shape of the CuO NPs. It can be used to visualize individual NPs and identify defects or agglomeration.^7^ Scanning electron microscopy (SEM) offers a lower resolution than that of TEM, but can be used to examine the surface morphology and topography of the CuO NPs.^6,156^ It is particularly useful for studying larger samples or for obtaining information about the particle distribution in a powder. Fourier transform infrared (FTIR) spectroscopy can be used to identify the functional groups present in the CuO NPs, such as the Cu–O bond. It can also be used to study the interactions between the NPs and any stabilizing or capping agents.

Ultraviolet-Visible (UV-Vis) Spectroscopy can be used to measure the optical properties of the CuO NPs, including their absorption and bandgap.^157^ It can be used to assess the electronic structure of the NPs and their potential applications in optoelectronic devices.^7^ Energy-dispersive X-ray spectroscopy (EDX) can be used to determine the elemental composition of the CuO NPs. By analyzing the X-rays emitted from the sample, EDX can confirm the presence of copper and oxygen in CuO NPs in terms of atom and mass percentage. This information is essential for understanding the stoichiometry of the material. Apart from that, the presence of other elements can be detected and quantified through EDX analysis. If integrated with the elemental mapping technique, the distribution of elements throughout a particular area of the NP can also be measured.^17^

The zeta potential measurement can also be employed for the assessment of the surface charge of the NPs. The value of zeta potential is crucial for understanding the stability of the NP dispersion and its interactions with other particles or surfaces.^6,158^ Atomic Force Microscopy (AFM) provides high-resolution images of the NP surface topography. It can reveal information about the size, shape, and surface morphology of the CuO NPs.^159,160^ Photoluminescence (PL) spectroscopy measures the emission of light from the NPs when they are excited by a light source. This technique can provide insights into the electronic structure and defect states within the CuO NPs.^161^ Thermogravimetric Analysis (TGA) determines the thermal stability of the NPs. By heating the sample and measuring the weight loss, TGA can identify the presence of any organic components or impurities.^156^ Raman spectroscopy provides information about the vibrational modes of the CuO NPs. This technique can help to identify the specific crystal structure and detect any phase transformations or defects.^162^

### 5.3. Influence of experimental parameters on CuO NP formation

The plant extract-mediated synthesis of CuO NPs is heavily influenced by various factors such as reaction conditions (pH, temperature, and time), precursor choice, and plant extract concentration. These parameters collectively determine the NP size, shape, and overall properties. By carefully controlling these variables, NPs with specific characteristics for a wide range of applications can be prepared.

#### 5.3.1. Effect of pH

The pH of the reaction medium significantly influences the formation of CuO NPs in plant extract-mediated synthesis. According to the literature, both alkaline and acidic conditions are responsible for the efficient formation of the CuO NPs. The work of Achamo *et al.* reports the *Artemisia abyssinica* leaf extract-mediated synthesis of CuO NPs, where pH values of 3 and 5 favored the formation of CuO NPs. However, at alkaline pH (higher than 7), the formation of CuO NPs was inhibited.^163^ The reason behind this can be explained by the fact that, at lower pH values, the phytochemicals present in the plant extract, such as polyphenols and flavonoids, tend to get protonated. This protonation enhances their reducing power, facilitating the reduction of copper ions to copper/copper oxide NPs. Additionally, the lower pH environment can promote the stability of the formed NPs by minimizing agglomeration. Conversely, at higher pH values, the phytochemicals may become deprotonated, reducing their reducing ability. This can hinder the formation of CuO NPs or result in the formation of larger, less stable NPs. Moreover, higher pH conditions can increase the solubility of copper ions, leading to the formation of copper hydroxide or other copper compounds instead of CuO NPs.^164^

On the contrary, studies in the literature also depict the effective formation of CuO NPs at higher pH values and report pH 7–9 as the optimum pH range.^165,166^ This may be because, when the pH falls below 7, creating acidic conditions, the effectiveness of the plant extract diminishes in acidic environments, which can result in the denaturation or degradation of the bioactive compounds responsible for the reduction and stabilization of the NPs. As a result, larger NPs tend to form under these conditions.^164,167,168^

#### 5.3.2. Effect of reaction temperature

The temperature significantly impacts the plant extract-mediated CuO NP synthesis. Generally, temperatures ranging from room temperature to 100 °C or slightly higher are employed for the synthesis of CuO NPs. Higher temperatures generally accelerate reaction rates, leading to smaller NPs due to rapid nucleation.^169^ However, plant extract degradation at extreme temperatures can limit the size reduction.^170^ While most green syntheses occur below 100 °C, some studies have shown that elevated temperatures can produce smaller NPs. For example, Sulaiman *et al.* achieved 20–50 nm CuO NPs at 100 °C using *Olea europaea* leaf extract.^171^ Similarly, *Eucalyptus globulus* leaf extract yielded 12 nm CuO NPs at 140 °C, compared to 68 nm at 30 °C.^172^ These findings suggest that the phytochemical composition of the plant extract, along with other factors, plays a crucial role in temperature-dependent NP formation. Post-synthesis heat treatments such as calcination and annealing can also influence the NP size.^7^ Higher annealing temperatures often result in larger NPs, as demonstrated in studies using black tea extract and henna extract.^173,174^ However, annealing can also lead to phase transitions and morphological changes, potentially affecting NP size and properties.^175^

#### 5.3.3. Effect of reaction time

Reaction time, or incubation period, significantly impacts the rate of NP formation and their resulting morphologies. Initially, the rate of NP nucleation and growth accelerates rapidly. However, after reaching an optimal point, the reaction rate stabilizes.^1^ When the incubation period is prolonged, the particles tend to form agglomerates and result in lager size.^176^ The optimum time for CuO NP synthesis using the plant extract of *Calotropis procera* was determined by considering the sharpest peak of the UV-Vis spectrum.^177^

#### 5.3.4. Effect of plant extract type and concentration

The type and concentration of plant extract used in the green synthesis of CuO NPs can significantly impact the properties of the resulting NPs.^178^ Studies have shown that different plant parts such as leaves, peels, fruits, and bark contain varying concentrations of phytochemicals such as flavonoids, tannins, terpenoids, and phenols that act as reducing and capping agents during CuO NP synthesis. For example, using a higher concentration of *Catha edulis* leaf extract (1/10 ratio) resulted in more uniform and defined spherical CuO NPs with less aggregation than lower extract concentrations (3/10 ratio), which showed some aggregation.^17^ Similarly, *Eucalyptus globulus* leaf extract was used to synthesize CuO NPs, demonstrating the effectiveness of leaves as a source of reducing agents.^19^ Citrus fruit peels have also been explored as they contain high amounts of phenolic compounds.^164^ The size, shape, and stability of the CuO NPs can be optimized by manipulating the plant extract type and concentration during the green synthesis process.

#### 5.3.5. Effect of precursor type and concentration

The synthesis of CuO NPs using plant extracts is significantly influenced by the type of copper precursor and its concentration. Different copper compounds such as copper nitrate and copper acetate can yield distinct morphologies and properties of CuO NPs due to their varying solubility and reactivity during the reduction process facilitated by plant biomolecules.^179^ Additionally, increasing the concentration of the precursor can enhance the reaction rate; however, it may also lead to agglomeration or a decrease in crystallinity if not optimized correctly.^180^ This delicate balance highlights the importance of selecting appropriate precursor types and concentrations to achieve desired NP characteristics for various applications including antimicrobial and antioxidant uses.

## 6. Textile effluent remediation by PEM-CuO NPs

The textile industry is a significant contributor to environmental pollution, primarily through the discharge of untreated or inadequately treated wastewater. This effluent is laden with various harmful substances that pose severe risks to aquatic ecosystems and human health.^181^ Textile manufacturing processes, particularly dyeing and finishing, are notorious for generating large volumes of wastewater.^182^ An estimated 200 liters of water are required to produce just one kilogram of cloth, and dyes, heavy metals, surfactants, and other toxic compounds contaminate a substantial portion of this water.^183^ The effluents often contain complex mixtures of pollutants, including organic and inorganic substances, which can severely degrade water quality. Studies indicate that textile dyeing alone accounts for approximately 20% of all freshwater pollution globally.^184^ While combating such pollution caused by textile dye-laden effluent, researchers utilized numerous photocatalysts and adsorbents. The PEM or simply the green-synthesized CuO NPs are potential materials to win such combats. There are a myriad of studies that report the efficacy of CuO NPs in the eradication of textile effluents.^15^ CuO usually removes toxic chemicals in two of the most preferred ways: adsorption, by their high surface area, and photocatalysis, by generating reactive oxygen species (ROS) when exposed to light.^185^ They are most preferred because of their cost-effectiveness and simple design.^186^ Adsorption by CuO NPs can be both physical and chemical surface interactions. It is an endothermic and spontaneous process. The adsorbent can be reused several times with high efficiency.^187^ The adsorption capacity depends on the degree of functionalization.^188^ CuO NPs can be activated under visible/solar light, ultraviolet (UV) light, and simulated sunlight.^189^Table 3 lists the treatment of various dyes in aqueous solutions by PEM-CuO NPs.

**Table 3:** PEM-CuO NPs for textile effluent remediation, particularly focusing on the removal of dyes, conditions of the process and results

Plant source	Plant part	Removal process	Conditions for maximum output	Removal efficiency	Ref.
*Justicia gendarussa*	Leaf	Photocatalysis	CuO = 20 mg, dye = 100 mL of 10 ppm, time = 5 h, under sunlight irradiation	97% of methylene blue (MB)	254
*Brassica rapa*	Leaf	Adsorption	CuO = 2.0 g L^−1^ at room temperature for 10 mg L^−1^ dye, time = 60 min	>92% of Amaranth dye, 80% Congo red (CR) and 47% Bismarck brown R (BBR)	255
*Eucalyptus globulus*	Leaf	Adsorption	Concentration = 0.04 g/50 mL, pH = 4.5, temperature = 25 °C	95 mg g^−1^*Q*_max_ (maximum adsorption capacity) for Methyl orange (MO)	19
*Punica granatum*	Leaf	Adsorption	CuO = 12 g L^−1^, time = 24 h, temperature = 298 K for 50 mg L^−1^ dye	95.80% of Safranin-O dye; *Q*_max_ = 189.54 mg g^−1^	256
*Ephedra alata*	Whole plant	Adsorption	CuO = 0.02 g, dye concentration = 10 mg L^−1^, pH = 7, temperature = 373 K	133.75 mg g^−1^*Q*_max_ for MB	257
*Seriphidium oliverianum*	Leaf	Photocatalysis	CuO = 10 mg for 10 mg L^−1^ dye, time = 60 min, using sunlight	65.231% ± 0.242 of Methyl green (MG) and 65.078% ± 0.392 of MO	21
*Psidium guajava*	Leaf	Photocatalysis	CuO = 0.1 mg mL^−1^, pollutant = 10 mL mixture of 1 mM, using visible light	91% of MB	258
				89% of Methyl red (MR)	
				80% of MO	
				97% of Eosin yellow EY)	
*Psidium guajava*	Leaf	Photocatalysis	CuO = 10 mg for both 40 ppm dye (20 mL), time = 2 h, irradiation under direct sunlight	93% of Nile blue, 81% of reactive yellow 160 (RY160)	259
Aloe-vera	Leaf	Photocatalysis	CuO = 25 mg, dye = 100 mL of 10 ppm, time = 24 min, using UV light	Maximum degradation of 96.1% of MO dye	260
*Ocimum tenuiflorum*	Leaf	Photocatalysis	CuO = 25 mg, dye = 100 mL of 10 ppm, pH = 4, using UV light (125 W)	96.4 ± 0.83% of MO dye	261
*Wedelia urticifolia*	Leaf	Adsorption	CuO = 10 to 40 mg, dye = 10 to 25 ppm, time = 0.5 h to 6 h, temperature = RT, stirring speed = 120 rpm, pH = solution pH	99% of RhB	262
*Aloe barbadensis*	Leaf	Adsorption	CuO = 0.05 to 0.5 g L^−1^, dye = 100 mg L^−1^, at pH = alkaline, time = 210 min, stirring speed = 150 rpm	98.89% of MB, *Q*_max_ = 95.5 mg g^−1^	263
*Ocimum americanum*	Leaf	Photocatalysis	CuO = 1 mg mL^−1^, dye = 0.1 mg mL^−1^, time = 200 min, using sunlight	75.69% of EY, 34.12% of Rhodamine 123 (Rh123), 71.06% of MB	264
*Carica papaya*	Fruit	Photocatalysis	CuO = 1 mg, dye = 10 mL of 0.001 M, time = 1 h	18.45% of MO under sunlight	265
				30.98% of MO under UV light	
Lemon tea extract	—		CuO = 1 mg, dye = 10 mL of 0.001 M, time = 1 h	31.95% of MO under sunlight	
				45.23% of MO under UV light	
*Annona muricata*	Leaf	Photocatalysis	CuO = 20 mg/100 mL of dyes, time = 1 h, using sunlight	90% of reactive red 120 (RR120)	266
				95% of MO	
*Ferulago angulate* (schlecht) boiss	Whole plant	Photocatalysis	CuO = 0.05 g, dye = 50 mL of 100 µM, time = 2.5 h, under fluorescent lamp (*λ* > 400 nm, 80 W)	84% of RhB	267
*Citrofortunella microcarpa*	Leaf	Photocatalysis	CuO = 10 mg L^−1^, dye = 10 ppm, pH = 5, time = 10 min, using UV light	98% of RhB	253
*Punica granatum*	Leaf	Adsorption	CuO = 0.4 g L^−1^, Equilibrium time = 120 min, temperature = 298 K	96% of Neutral red (NR) dye; *Q*_max_ = 283 mg g^−1^	268

## 7. Pharmaceutical waste remediation by PEM-CuO NPs

Pharmaceutical waste encompasses a variety of antibiotics, painkillers, and anticancer drugs that pollute the environment and disrupt the natural functioning of ecosystems.^190^ The increase in environmental pharmacological substances and their potential harmful effects on biological systems are global issues, especially challenging for countries with high population growth. Evidence shows that these substances threaten genetic, species, and community diversity^191^ and cause chronic damage, behavioral changes, accumulation in tissues, reproductive damage, and inhibition of cell proliferation, and can be incorporated into food chains.^192^ Plants can accumulate pharmaceutical compounds in their seeds, roots, and leaves. This accumulation has been linked to a reduction in grain yield by up to 50%. Once in water, these compounds can alter the behavior of fish and other organisms, inhibit reproduction in crustaceans, and even cause the death of exposed organisms.^193^

The application of NPs for the eradication of these pharmaceutical wastes is previously reported in the literature.^194–196^ Photocatalytic degradation and adsorptive removal are more predominant than other removal techniques. However, the exploitation of PEM-CuO NPs for such cause is a newer concept, and previous reports show its potential applicability.^197^ In Table 4, we compile the application of PEM-CuO NPs and their composites for the treatment of pharmaceutical waste.

**Table 4:** Pharmaceutical waste treatment using PEM-CuO NPs and their composites

Plant source	Plant part	Precursor of CuO NPs	NP name	Pharmaceutical waste	Treatment process	Treatment efficiency	Ref.
*Ocimum sanctum*	Leaf	Copper acetate	CuO	Doxycycline hydrochloride	Adsorptive removal	*Q*_max_ = 8.56 mg g^−1^ at 100 mg L^−1^, 170 min, pH 6 at 298 K	197
*Platanus occidentalis*	Leaf	Copper sulphate	CuO	Paracetamol	Adsorptive removal	*Q*_max_ = 64.52 mg g^−1^	269
Green tea extract	Leaf	Copper sulphate	CuO	Ciprofloxacin	Adsorptive removal	92% (con. 0.01 mg L^−1^, 0.75 g L^−1^, pH 4, 180 min	
*Tragia involucrata*	Leaf	Copper nitrate trihydrate	g-C_3_N_4_/CuO	Ciprofloxacin	Photodegradation	93%	270
*Parthenium hysterophorus*	Whole plant	Copper(ii) sulphate	CuO	Rifampicin	Photodegradation	98.43% (65 °C, 50 mg dosage, 10 mg L^−1^ drug, pH 2, time 8 min	271
*Euphorbia polygonifolia*	Aerial parts	Copper(ii) sulfate pentahydrate	Fe_3_O_4_@CuO	Metronidazole	Photodegradation	89%	272
				Ciprofloxacin		94%	
				Cephalexin		96%	
*Psidium guajava*	Leaf	Copper sulphate	CuO/Fe_2_O_3_	Tetracycline	Photodegradation	88% in 80 min	273
*Macadama* nut shell extract	Nut shell	Copper nitrate hexahydrate	CuO NPs	Tetracycline	Photodegradation	74% in 120 min	274
*Ferula persica*	—	Copper sulphate	CuO–CdO-bentonite	Levofloxacin	Photodegradation	96.11% at catalyst dosage = 0.4 g L^−1^, concentration = 10 mg L^−1^ and pH = 8	275

## 8. Mechanism of pollutant eradication by PEM-CuO NPs through photodegradation and adsorption

CuO NPs have been used as potential photocatalysts in the degradation of organic pollutants including toxic dyes and pharmaceutical wastes due to their unique properties including high surface area, adjustable bandgap and good photocatalytic characteristics. This photodegradation process mainly involves the generation of reactive oxygen species (ROS) under light irradiation, which subsequently break down the pollutants.

Upon irradiation with ultraviolet (UV) light or visible light, the energy of the incident photons can excite the electrons of CuO NPs from their valence band (VB) to the conduction band (CB), generating electron–hole pairs.^198,199^ These holes serve as powerful oxidizing agents and oxidize water molecules to generate ROS such as hydroxyl radicals (OH˙^−^). At the same time, the electrons in the conduction band can reduce the adsorbed oxygen on the CuO NP surface to form superoxide radicals (O_2_˙^−^). These ROS decompose the molecules of organic pollutants such as dyes and pharmaceutical wastes through oxidation reactions.^200–203^ Once the molecules of organic pollutants are attached to the surface of CuO NPs, the ROS species bind the molecule. This breaks the chemical bond of organic molecules and ultimately degrades the toxic pollutants into less harmful products such as carbon dioxide and water. The probable reaction mechanism for the photodegradation of toxic organic pollutants using CuO NPs as photocatalysts is as follows:CuO + *hν*→ CuO (e_cb_^−^ + h_vb_^+^)H_2_O + h^+^ → H^+^ + OH˙^−^O_2_ + 2e^−^ → O_2_˙^−^O_2_˙^−^ + H^+^ → HO_2_˙^−^HO_2_˙^−^ → H_2_O_2_ + O_2_H_2_O_2_ → 2OH˙^−^OH˙^−^ + organic pollutants → degradation products (CO_2_ + H_2_O)

**Fig. 6:**
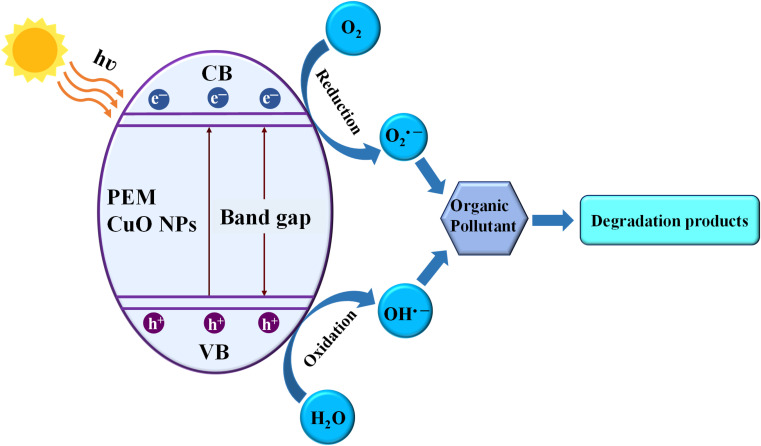
Basic mechanism of the photocatalytic activity of CuO NPs for degrading organic pollutants.

CuO NPs show promising efficiency for the photodegradation of toxic organic dyes and pharmaceutical wastes due to their excellent photocatalytic properties. While the formation of ROS and the oxidation of organic pollutants are crucial to the degradation process, other factors such as particle size and concentration of NPs, bandgap energy, solution pH, temperature and reaction time also affect the photocatalytic degradation activity.^204^ The pH of the solution plays a significant role in the adsorption capacity and degradation efficiency. At lower pH values, CuO NPs typically carry a positive charge, which enhances their interaction with negatively charged molecules of pollutants. However, at higher pH values, the surface becomes negatively charged, which can repel negatively charged pollutants and reduce the degradation efficiency. Moondeep *et al.* have reported a similar phenomenon, in which, at higher pH values, the CuO NP surface is negatively charged, which improves its interaction with cationic dyes. Meanwhile, at lower pH values, the positively charged surface of CuO NPs enhances their interaction with anionic dye.^205^ Along with the pH of the solution, the concentration of CuO NPs in the reaction mixture also plays a crucial role in photocatalytic efficiency. An adequate concentration of CuO is necessary to optimize the light absorption and pollutant interaction. A much lower concentration may not provide sufficient active sites for adsorption, while too high concentrations can cause turbidity that can lead to light scattering and reduced effective surface area for reactions.^206^ Moreover, the temperature and time can influence the photocatalytic activity to some extent. At higher temperatures, the reaction rates increase with the increase in molecular collisions. However, excessively high temperatures can reduce dissolved oxygen concentration, leading to increased electron–hole recombination, which hinders photocatalytic activity.^207^ Additionally, optimal irradiation times allow sufficient generation of ROS, facilitating the degradation process. After reaching a plateau at a favorable time, further exposure does not significantly enhance the degradation efficiency.^206^ Therefore, it is necessary to optimize these parameters to improve the photocatalytic activity. Fig. 6 illustrates the underlying mechanism of photocatalysis by PEM-CuO NPs.

The high surface area and surface charge of CuO NPs contribute to their adsorption capabilities along with the photocatalytic degradation capacity. This mechanism of adsorptive removal mainly involves the interaction between the toxic organic pollutant molecules and the CuO NPs in different physicochemical ways. The surface charge of CuO NPs can vary with pH, which influences the interaction of CuO NPs with the pollutant molecules. If the pollutant molecules are charged, they can be attracted to the oppositely charged surface of the CuO NPs through electrostatic forces. At higher pH levels, negative charges on CuO surfaces increase, which enhances the electrostatic attraction with cationic pollutant molecules, whereas, at lower pH levels, the positively charged surface of CuO NPs attracts the anionic pollutant molecules.^208^ Moreover, the capping or stabilizing molecules on PEM-CuO NPs may contain plant phytochemicals and the functional groups present in them contribute to toxic pollutant molecule adsorption through hydrogen bonding and van der Waals interactions.^209^ Furthermore, the aromatic hydrocarbons present in organic pollutants can facilitate the adsorption procedure by interacting with the π-electron cloud of the CuO NPs through π–π interactions.^210^ Similar to photodegradation, the organic pollutant adsorption process is influenced by the concentration of pollutants and the available active sites on the CuO NPs.

## 9. Antimicrobial activity of PEM-CuO NPs

The concept of antimicrobial activity involves the ability of certain agents to kill or inhibit disease-causing microbes. This activity can be exhibited by various antimicrobial agents including those that are antibacterial, antifungal, or antiviral.^211^ Metal NPs, especially those of silver, gold, and copper, have been thoroughly investigated for their antibacterial properties, and the results indicate that they are quite successful in treating a wide range of bacterial types.^212^ Inorganic metal oxide NPs such as CuO, ZnO, MgO, TiO_2_, and SiO_2_ have significant antimicrobial features.^213^ CuO NPs have demonstrated broad-spectrum antibacterial activities against a range of bacteria as well as antifungal activities.^214^Table 5 and Table 6 provide data on the antibacterial and antifungal activities of PEM-CuO NPs, respectively.

**Table 5:** Antibacterial activity of PEM-CuO NPs against various bacterial strains

Bacterial strain and type	Plant source	Dosage^a^ of CuO NPs	Antimicrobial efficiency in terms of zone of inhibition (ZOI)	Ref.
*Bacillus subtilis* (Gram-positive)	*Morinda citrifolia*	25 µL	13.6 ± 1.1 mm	168
	*Pterolobium hexapetalum*	50 µg mL^−1^	15 ± 0.29 mm	242
	*Abutilon indicum*	5 mg	15 ± 0.11 mm	276
	*Allium sativum*	150 µg mL^−1^	10.90 ± 0.62	277
	*Portulaca oleracea*	200 µg mL^−1^	16.7 ± 0.6 mm	278
*Escherichia coli* (Gram-negative)	*Averrhoa carambola*	100 µg mL^−1^	24 mm	6
	*Ailanthus altissima*	100 µg mL^−1^	18 mm	279
	*Malus domestica*	100 µg mL^−1^	17 mm	280
	*Aerva javanica*	50 µg mL^−1^	5 ± 1 mm	281
	*Ruellia tuberosa*	75 µg mL^−1^	∼11 mm	282
*Staphylococcus aureus* (Gram-positive)	*Averrhoa carambola*	100 µg mL^−1^	16 mm	6
	*Malus domestica*	100 µg mL^−1^	19 mm	280
	*Ruellia tuberosa*	75 µg mL^−1^	∼18 mm	282
	*Cardiospermum halicacabum*	900 µg mL^−1^	22.6 ± 1.5 mm	283
	*Cordia sebestena*	1000 µg mL^−1^	24 mm	284
*Salmonella typhi* (Gram-negative)	*Averrhoa carambola*	100 µg mL^−1^	16 mm	6
	*Moringa oleifera*	—	∼12.5 mm	285
	*Solanum melongena*	150 µg mL^−1^	15 mm	286
	*Falcaria vulgaris*	0.5 mg mL^−1^	18 ± 0.4 mm	287
	*Aloe vera*	1 mg mL^−1^	13 ± 0.02 mm	288
*Streptococcus pneumoniae* (Gram-positive)	*Psidium guajava*	80 µg mL^−1^	∼15 mm	16
	*Citrus reticulata*	50 µg mL^−1^	18 mm	289
	*Citrus sinensis*		27 mm	
	*Citrus limon*		17 mm	
	*Antigonon leptopus*	50 µg/50 µL	24 mm	290
*Klebsiella pneumonia* (Gram-negative)	*Azadirachta indica*	—	∼35 mm	291
	*Simmondsia chinensis*	—	∼25 mm	
	*Mentha spicata*	100 µg mL^−1^	17.4 ± 0.6 mm	292
	*Allium sativum*	150 µg mL^−1^	10.65 ± 0.63 mm	277
	*Cassia fistula*	1 mg	34 ± 1.59 mm	293
	*Melia azedarach*	1 mg	39 ± 2.00 mm	
*Staphylococcus epidermidis* (Gram-positive)	*Fumaria indica*	5 mg/5 mL	3.98 mm	294
	*Zanthoxylum armatum*	100 µL	11.66 ± 0.33 mm	295
	*Zanthoxylum armatum*		19.66 ± 0.33 mm	
	*Berberis lycium*		16.66 ± 0.33 mm	
	*Momordica charantia*	1.25 mg/50 µL	23 ± 2.0 mm	
*Pseudomonas aeruginosa* (Gram-negative)	*Adhatoda vasica*	1 mg mL^−1^	12 mm	296
	*Ficus religiosa*	1 mg mL^−1^	13 mm	297
	*Leucas aspera*	500 µg	10 mm	298
	*Morinda tinctoria*	500 µg	10 mm	
	*Acanthospermum hispidum*	500 µg mL^−1^	19 mm	299

a Dosage for which highest ZOI found was selected for inclusion.

**Table 6:** Antifungal activity of PEM-CuO NPs against various fungal strains

Fungal strain	Plant source	Dosage	Antifungal efficiency	Ref.
*Aspergillus niger*	*Manilkara hexandraf*	10 µL mL^−1^	a9 mm	300
	*Malus domestica*	0.05 mgL^−1^	b92%	301
	*Cissus quadrangularis*	1000 ppm	b∼85%	302
	*Syzygium alternifolium*	40 µg mL^−1^	a∼7 mm	303
	*Tinospora cordifolia*	100 µg mL^−1^	a∼11 mm	304
	*Brassica oleracea*	100 µg mL^−1^	a9 mm	305
	*Bougainvillea*	5 mg mL^−1^	a∼19 mm	306
	*Eichhornia crassipes*	100 µg mL^−1^	a18.33 ± 1 mm	248
*Candida albicans*	*Tinospora cordifolia*	100 µg mL^−1^	a∼15 mm	304
	*Ephedra alata*	6 mg mL^−1^	a16.2 mm	307
	*Allium sativum*	150 µg mL^−1^	a10.05 ± 0.63 mm	277
*Aspergillus flavus*	*Allium sativum*	150 µg mL^−1^	a9.30 ± 0.58 mm	277
	*Spinacia oleracea*	—	a∼14 mm	308
	*Eichhornia crassipes*	100 µg mL^−1^	a∼16 mm	248
	*Cissus quadrangularis*	1000 ppm	b∼85%	302
*Fusarium oxysporium*	*Eichhornia crassipes*	100 µg mL^−1^	a∼18 mm	248
	*Heliotropium bacciferum*	100 µg mL^−1^	b71.11%	309
	*Tamarix aphylla*	100 mg L^−1^	b88%	310
*Aspergillus fumigatus*	*Allium sativum*	150 µg mL^−1^	a9.95 ± 0.65 mm	277
	*Eichhornia crassipes*	100 µg mL^−1^	a∼14 mm	248
*Saccharomyces cerevisiae*	*Ephedra alata*	6 mg mL^−1^	a18.4 mm	307
*Fusarium culmorum*	*Eichhornia crassipes*	100 µg mL^−1^	a∼22 mm	248
*Botrytis cinerea*	*Heliotropium bacciferum*	100 µg mL^−1^	b50.74%	309
*Rhizoctonia solani*	*Heliotropium bacciferum*	100 µg mL^−1^	b81.48%	309

a Zone of inhibition (ZOI).

b Percentage of inhibition; the dosage for which the highest efficiency was found, was selected for inclusion.

## 10. Mechanism of the antimicrobial activity of PEM-CuO NPs

Recently, NPs have received immense attention for various biomedical applications, especially as antimicrobial agents. A broad spectrum of microorganisms have been reported to be susceptible to different NPs. CuO NPs have emerged as promising antimicrobial agents due to their salient physicochemical properties such as large surface area, small particle size, stability, electrostatic attraction, hydrophobic interactions and van der Waals forces. In addition, the abundance of copper makes it quite cheap to produce CuO NPs on a large scale. Trissa *et al.* synthesized CuO NPs using the leaf extract of *Averrhoa carambola* and investigated their antibacterial activity against Gram-positive and Gram-negative bacterial strains.^6^ Vijay *et al.* synthesized CuO NPs utilizing *Aloe vera* leaf extract and explored their bactericidal activity against fish pathogens.^215^ Both of the studies found prominent antimicrobial activity.

While the precise mechanism for the antimicrobial activity of PEM-CuO NPs is elusive, several studies have proposed that the antimicrobial property is primarily attributed to the interaction of the NPs with the outer layer of the microbial cell wall. Some studies reported in the literature suggested that the Cu^2+^ ions released from CuO NPs bind to the negatively charged cell membrane due to electrostatic and van der Waals forces.^216,217^ This interaction eventually led to the formation of the pits by disrupting the integrity of the cell membrane. The produced Cu^2+^ enters the cell and damages the nucleic acid of DNA molecules as well as hampers cell processes such as enzyme activity and metabolism, which eventually lead to cellular breakdown. Other studies proposed that the generation of reactive oxygen species (ROS) is the primary initiator of antimicrobial activity. ROS can oxidize cell components by damaging DNA, proton efflux pumps and proteins. It can also cause enzyme breakdown, reduce the quantity of the antioxidant glutathione, and ultimately cause cell death.^218–220^

**Fig. 7:**
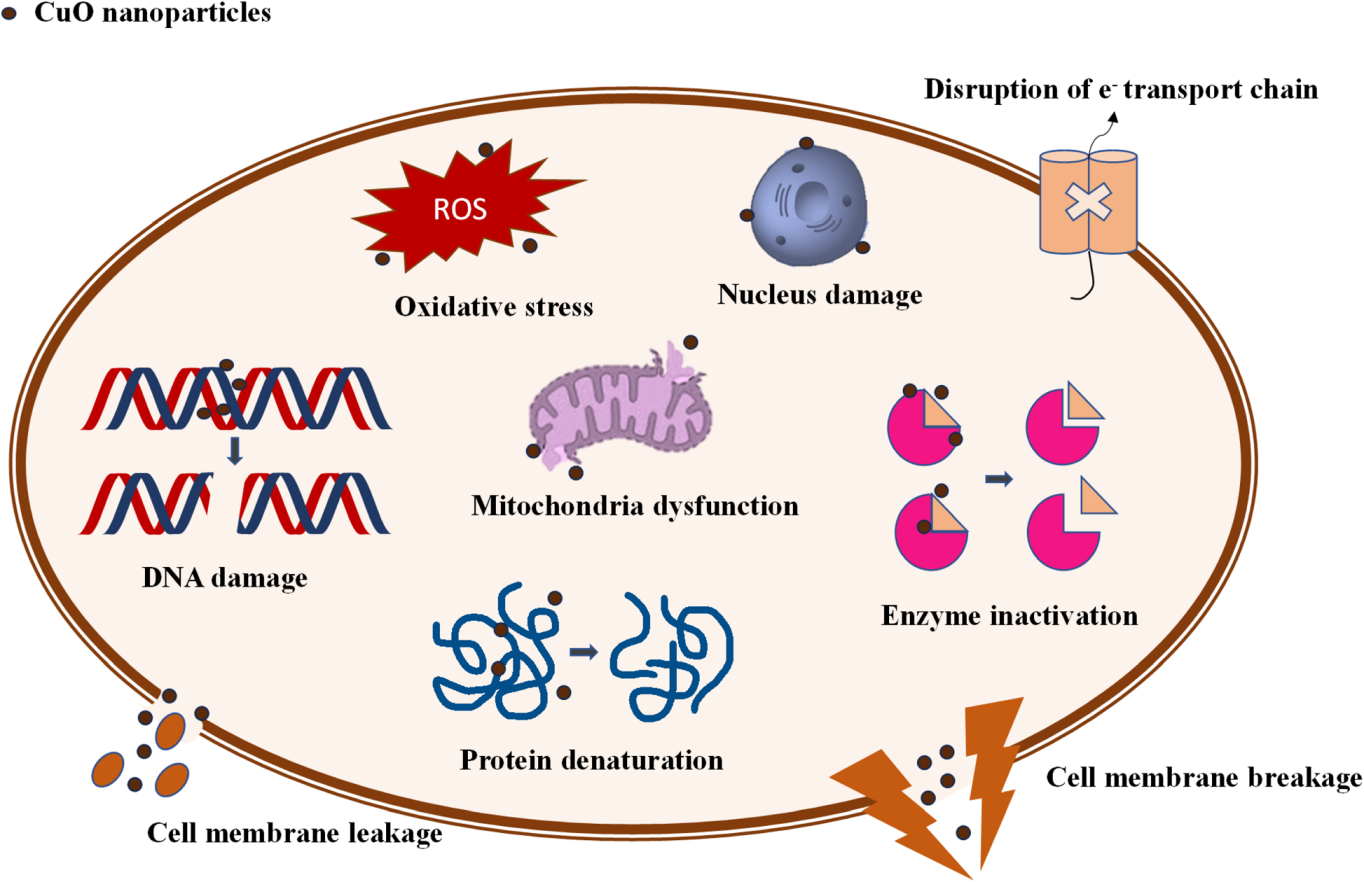
Probable mechanism of the antimicrobial activity of PEM-CuO NPs.

Another probable mechanism is the electrostatic adhesion of CuO NPs to the cell membrane of microbes. When CuO NPs get attached to the cell, the shape of the plasma membrane changes and causes the leakage of internal cellular components, resulting in cell death. Along with these proposed mechanisms, the size, shape, and concentration of NPs affect the antimicrobial activity significantly. Fig. 7 shows the probable mechanism of antimicrobial activity of CuO NPs. The antimicrobial mechanism involving membrane disruption and ROS generation is quite ubiquitous for both bacteria and fungi. However, the distinct cell wall structures and metabolic processes of bacteria and fungi tailor the mechanism differently. The bacterial cell wall is made up of peptidoglycan, while the fungal cell wall is composed of chitin and glucan. These differences in the cell wall structure affect the mode of action of CuO NPs. In the case of bactericidal activity, CuO NPs attached to the bacterial cell wall damage the cell structure and interact with DNA, causing cell damage and preventing cell replication. In contrast, for fungicidal activity, CuO NPs inhibit fungal growth by damaging the mycelial structure and interacting with fungal enzymes, which disrupt the cellular processes.

## 11. Challenges and limitations in PEM-CuO NPs synthesis

Although PEM-CuO NPs offer a sustainable as well as environment-friendly alternative to conventional methods, it also faces some challenges and limitations.

### 11.1 Difficulty in controlling size-shape-morphology and reproducibility concerns

The most crucial challenge faced by such synthesis procedure is the difficulty in controlling the size, shape and morphology of the synthesized CuO NPs due to the complexity of the plant extracts.^221^ Plant extracts are complex mixtures of various biomolecules that play crucial roles in the synthesis CuO NPs by reducing metal ions, preventing aggregation, and controlling nanoparticle growth. However, the exact composition and concentration of these biomolecules can vary significantly depending on several factors such as environmental conditions during plant growth, temperature, light, water availability, and nutrient levels.

Furthermore, extraction methods as well as the choice of solvents, temperatures, and extraction times can significantly impact the types and amounts of compounds extracted.^164^ This variability in composition can make it difficult to precisely control the size, shape, and morphology of the synthesized CuO NPs. One of the biggest challenges in reproducibility is that even minor changes in size, shape, or nanoparticle surface chemistry can significantly affect their stability, interactions with biological media, and overall biodistribution.^222^ Additionally, the complex interactions between different biomolecules and synthesis parameters pose challenges in optimizing the synthesis process, and the presence of residual organic molecules from the plant extract can further complicate the characterization of the resulting CuO NPs.^223^

### 11.2 Hurdles in optimizing the reaction parameters

The optimization of reaction parameters for the PEM NP synthesis is a monumental task as it requires careful control of several key parameters for achieving desired properties of NPs. For example, the precursor salt concentration of CuO NPs directly impacts the availability of copper ions for reduction. Higher concentrations can accelerate the nucleation and growth, which leads to the formation of larger particles. On the contrary, lower concentrations result in slower growth and smaller particles.^5^ Thus, balancing sufficient copper ions for NP formation with the risk of uncontrolled growth and aggregation is very crucial.

The pH of the reaction mixture influences the redox potential of reducing agents in the plant extract and the surface charge of the resulting CuO NPs.^224^ This affects the reduction rate, NP stability, and morphology. Therefore, the adjustment of pH with acid and base requires careful consideration for specific plant extract and the desired CuO NP properties. Temperature significantly affects the reaction kinetics. Although it is understood that a higher temperature accelerates reduction, excessively high temperatures result in rapid and uncontrolled growth, yielding larger and less uniform particles.

The reaction time regulates the exposure duration of copper ions to the reducing agents. Insufficient time may lead to incomplete reduction, while excessive time can result in overgrowth or aggregation. Balancing the plant extract concentration is also essential for controlling size and preventing aggregation. Higher concentrations can accelerate reduction and improve stabilization but may also increase nucleation sites, potentially leading to smaller particles. The stirring rate ensures uniform reactant distribution and promotes efficient mixing, enhancing the uniformity of NP formation. Optimal stirring ensures proper mixing without excessive shear forces that could affect NP growth.^225^

### 11.3 Difficulty in mass production

Industrial production of PEM-CuO NPs is a very convoluted task due to challenges in maintaining consistent plant extract quality (due to natural variations), ensuring uniform mixing and reaction conditions in larger reactors, managing mass and heat transfer limitations, implementing sophisticated process control and monitoring, addressing economic factors, guaranteeing reproducibility, and complying with regulations. Controlling the inherently variable plant extracts, scaling reactor design, and maintaining consistent product quality at a larger volume are also the key hurdles to overcome for successful industrial production of CuO NPs using this green synthesis method.^226^

### 11.4 Limitation in product lifespan and consistency

The PEM-CuO NPs face stability and shelf-life challenges due to residual organic matter, which may originate from the biomolecules in the plant extract. This residue can oxidize or degrade, resulting in an inconsistent production. Additionally, the inherent tendency of NPs to aggregate, undergo Ostwald ripening, and experience surface degradation, particularly in the case of CuO due to factors such as oxygen, moisture, and temperature, further degrades the material. The pH of the storage medium, the choice of solvent, and exposure to light also contribute to this problem. Preventive measures include thorough purification to remove organic residues, surface modification with stabilizing agents, controlled storage in inert atmospheres at low temperatures away from light, optimizing pH and solvent, and encapsulation within protective matrices can ensure better stability but upsurges the cost.^227^

### 11.5 Toxicity and environmental safety concerns

Although the plant extract-mediated route for CuO NP synthesis eliminates the need for toxic substances as reducing or stabilizing agents, the CuO NPs themselves can still pose toxicity and environmental risks. CuO NPs in general can cause harm through oxidative stress, copper ion release, and direct physical interaction with cells. The PEM-CuO NPs raises concerns due to the presence of potentially toxic residual organic components and possible allergens coming from the plant extract. When compared with the chemically synthesized counterpart, the PEM-CuO NPs exhibit lower toxicity. The work of Saif *et al.* demonstrated that chemically synthesized CuO NPs show a much higher toxicity, with an EC_50_ value of 0.102 ± 0.019 mg L^−1^, while PEM-CuO NPs show an EC_50_ value of 0.69 ± 0.226 mg L^−1^, with more than five times lower toxicity.^228^ This difference may have caused by the plants producing stable NPs, where phenolic compounds such as flavonoids or tannins act as stabilizing and coating agents, which leads to lower dissolution of PEM-CuO NPs than chemically synthesized CuO NPs.

To reduce the toxic effects of PEM-CuO NPs, optimizing the NP concentration, selection of appropriate dosage, considering real environmental conditions while measuring dissolution, encapsulation, and composite making with magnetic nanocomposites should be considered.^229,230^

## 12. Conclusion and future prospects

The future of PEM-CuO NP synthesis is promising, especially considering increasing environmental concerns. PEM-CuO NP synthesis offers a sustainable and environmentally friendly approach to nanomaterial production methodology, where plant materials act as reducing agents and limit the use of toxic chemicals needed in the conventional synthesis procedures. These NPs exhibit unique properties including small particle size, high surface area, stability, excellent catalytic activity and notable antimicrobial properties, making them suitable for various applications including dye degradation, water purification, drug delivery, antimicrobial activity and even tissue engineering.

However, it is crucial to address the toxicological aspects of these NPs to ensure their safe applications in diversified fields. As there is no established evidence regarding their toxicity, further *in vitro* and *in vivo* research is necessary to understand the mechanisms behind any kind of adverse effects. Considering the potential functionalities of CuO NPs, it is imperative to prioritize safety by conducting thorough toxicological assessments. Researchers can focus on these areas to enhance the viability and safety of plant-based NP synthesis and ultimately contribute to the advancement of nanotechnology. Further, this review article can provide a fundamental understanding of plant-mediated NPs to aid in their research.

## Data availability

This review article does not contain any original data generated or analyzed by the authors.

## Author contributions

M. Bin Mobarak: conceptualization, methodology, writing – original draft, review & editing, supervision; MD. F. Sikder: writing – original draft; K. Muntaha: writing – original draft; S. Islam: writing – original draft; S. Rabbi: writing – original draft; F. Chowdhury: supervision, writing – original draft, review & editing.

## Conflicts of interest

There are no conflicts to declare.
